# Orientation driven design and mechanical optimization of gyroid TPMS lattice structures

**DOI:** 10.1038/s41598-026-35201-5

**Published:** 2026-01-29

**Authors:** Mohamed S. El-Asfoury, Nehal E. El-Bedwehy, Mostafa Shazly, Ahmed Elkaseer

**Affiliations:** 1 Production Engineering and Mechanical Design Department, Faculty of Engineering, Port Said University, Port Fouad, 42526 Port Said Egypt; 2 Mechanical Engineering Department, Faculty of Engineering, The British University in Egypt, El-Sherouk City, 11837 Cairo Egypt

**Keywords:** Triply periodic minimal surfaces (TPMS), Gyroid lattice structures, Structural orientation, Mechanical properties, Finite element analysis (FEA), Fused filament fabrication (FFF), Mechanical engineering, Polymers

## Abstract

Triply Periodic Minimal Surface (TPMS) lattice structures, particularly Gyroid morphologies, are gaining attention for their high specific strength, energy absorption, and geometric adaptability. However, the large body of prior studies has focused on comparing different TPMS topologies or materials, with limited attention to how the build orientation of a single Gyroid structure influences its mechanical behavior. Addressing this gap, this study presents a novel design strategy by systematically varying the build orientation of Gyroid structures fabricated via Fused Filament Fabrication (FFF) using PLA-metal composite. Six models, termed G0 - G5, were created by altering orientation angles relative to the z-axis. Experimental and finite element analyses showed strong agreement and revealed that axially aligned models (G1, G3, G5) achieved significantly higher stiffness, strength, and energy absorption. Homogenization confirmed orientation-dependent anisotropy, and true stress analysis based on CAD-derived cross-sections improved stress accuracy. Increasing wall thickness induced a shift from bending- to stretch- and shear-dominated deformation. Power-law fitting of mechanical properties versus relative density achieved R² > 0.95, validating Gibson-Ashby scaling. These insights support tailored Gyroid designs for crashworthiness, aerospace, automotive and biomedical applications.

## Introduction

In a wide range of research domains aimed at mechanical applications, biological, and even aerospace applications, cellular solids demonstrate lightweight and superior energy absorption capability^1,2^. Due to their numerous benefits, including low weight with high specific strength, stiffness, and remarkable energy absorption capabilities, lightweight cellular materials have become widely used^3^. Triply Periodic Minimal Surfaces (TPMS) represent a promising class of open-cell cellular materials that have garnered considerable attention in recent years due to their exceptional mechanical and physical properties, coupled with mathematically controllable geometric topology. The essential characteristic of TPMS structures—featuring smooth surfaces with zero mean curvature—effectively mitigates stress concentration phenomena, making them particularly advantageous for engineering applications. TPMS structures exhibit several desirable properties, including high porosity, a large specific surface area, superior specific strength and stiffness, exceptional energy absorption, and fatigue resistance^4^. For a wide range of applications, TPMS metamaterials represent an excellent area of study. They are relevant in various disciplines, including thermodynamics (heat sinks)^5^, piezoelectricity^6^, permeability mechanics, elastic-plastic mechanics, acoustics (sound absorption, sound insulation, and vibration reduction)^7–9^, biomedical applications, ^10,11^, among others. By integrating materials, lattice morphologies, volume fractions, and pore sizes, it is possible to create diverse patterns for various applications^12^. These advancements pave the way for lattice exploration, enabling the production of lattice structures with properties that surpass those of traditional engineering materials.

The Gyroid lattice structure, a representative form within the class of TPMS lattice structures, is inspired by biological microstructures, particularly those found in butterfly wings^13^. It is distinguished by its exceptional specific strength coupled with low density. Additionally, the gyroid structure offers a comparatively larger connection area than other prevalent TPMS architectures, such as the Schwarz Diamond and Primitive structures, rendering it especially advantageous for fabricating graded cellular materials^14,15^. Yang et al. designed Gyroid cellular structures with density gradients varying from 20% to 10% along specific axes, demonstrating enhanced energy absorption capacities and improved large-strain deformation protection before densification compared to uniformly structured counterparts^16^. Furthermore, recent research highlights the Gyroid structure’s significant potential in optimizing energy absorption performance across diverse engineering applications^17^. Consequently, advancing the mechanical properties of Gyroid structures remains critically important.

According to the precision of design of TPMS structures, the additive manufacturing process (AM) is a prime manufacturing processing candidate to produce these structures. It can produce customized designs with a complex curvature, in one operation, with high resolution, and using a wide range of materials^18^. One of the popular AM techniques is Fused Filament Fabrication (FFF). The materials used for this technique are mainly thermoplastics such as acrylonitrile butadiene styrene (ABS) and polylactic acid (PLA). Thermoplastic extrusion technologies are now affordable due to their simplicity and the availability of safe and inexpensive materials. In addition, the FFF technique enables fabrication of complex lattice structures using various thermoplastic materials, making it particularly suitable for producing TPMS structures with controlled mechanical properties for diverse engineering applications^19^.

Several studies have been carried out to examine the mechanical properties of TPMS structures. Maskery et al. stated that the number of unit cells in TPMS lattice structures causes an increase in the elastic modulus. It is crucial to note that the applied loading orientation can alter the elastic modulus of the TPMS structure. The orientation of the TPMS geometry is a vital parameter for enhancing the elasticity of the final structure^20^. Abueidda et al. ^21,22^ analyzed the mechanical performance of TPMS structures through experimental and numerical simulation methods. By comparing the mechanical properties of Gyroid, Primitive, an I-graph-wrapped package (IWP)-TPMS structures, it is shown that Gyroid structures have the best energy absorption performance. Also, it achieves the highest strength compared to primitive, IWP, and Neovius structures. The gyroid structure with PLA or carbon fiber/PLA material exhibits better compression and energy absorption^23^. Additionally, Gyroid structures with lower densities show a comparatively reduced elastic modulus with stretched behavior^24^. According to Feng et al.^2^, the strength of the Diamond and Gyroid structure under dynamic loading was higher than the static results, which indicated that the structures present certain strain rate sensitivity. Under quasi-static loading, the Gyroid structure mostly experiences X-shaped deformation, with the shear deformation zone dispersed along the diagonal of the specimen. Rather, the diamond structure’s deformation behavior displays localized shear collapse characteristics, and the specimen’s 45◦ direction is mostly where the shear-like deformation localization region is found. The self-supporting nature of the Gyroid structure with its original form, renowned for its remarkable strength and energy absorption, makes it ideal for 3D printing applications. One of these promising applications is a helmet that was presented by Chao Bao et al.^25^. Also, Gyroid structures offer a homogeneous temperature distribution that makes them suitable for heat exchangers and thermal management in electronics cooling^26^. According to earlier research, the gyroid structure exhibits attractive mechanical characteristics. Besides, the altering of the main parameters is still a challenging task to optimize the mechanical requirements according to the application.

**Fig. 1:**
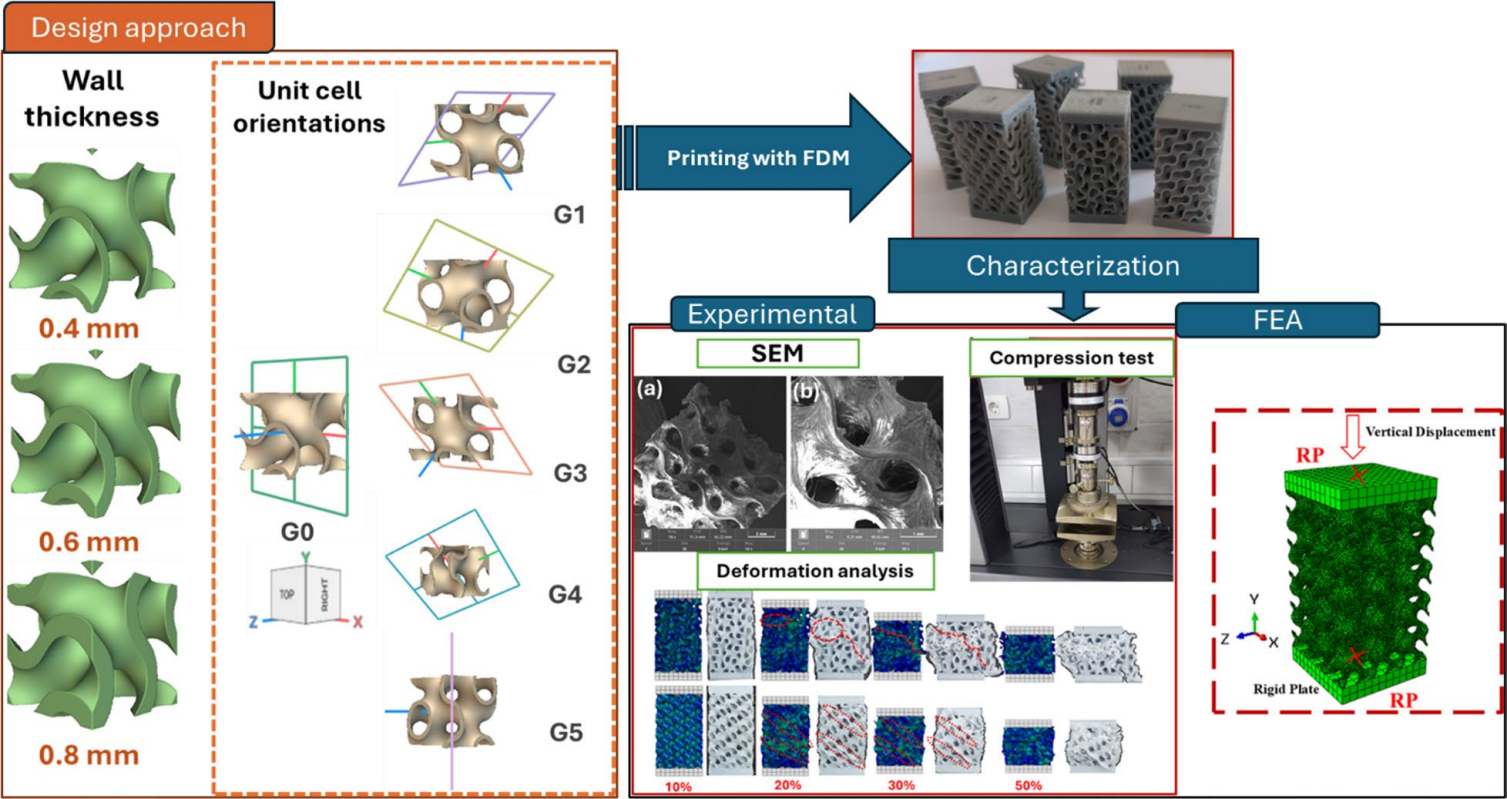
Graphical illustration of the study methodology sequence.

Unlike most previous studies that either compared different TPMS topologies or investigated a single build orientation, the present work establishes a systematic orientation-driven design and validation framework for a single Gyroid architecture. Six distinct orientations (G0-G5) were parametrically generated to cover in-plane, out-of-plane, and compound rotations, enabling a quantitative assessment of orientation-induced anisotropy. The originality of this study lies in integrating experimental compression testing, finite element modeling, and directional homogenization to correlate build orientation with stiffness and energy absorption. Furthermore, by employing CAD-derived cross-sectional analysis to calculate true stress, the study ensures direct linkage between design geometry and as-printed performance. This integrated methodology advances the understanding of how additive-manufactured Gyroid lattices can be tailored for orientation-specific mechanical optimization across industrial and biomedical applications. Figure 1 shows the sequence of the present study.

## Methodology and experimental work

### Design of the oriented gyroid

TPMS lattice structures are defined with three parameters: unit cell type, unit cell size, and wall thickness. As a result, altering one or more of these factors will significantly alter the designed structure. The impact of unit cell orientation and wall thickness on the mechanical characteristics of the developed structures has been investigated in this study. For every design, a new layout is produced by changing the plane angle of the building unit cell about the z-axis. All lattice structures were generated using nTopology software. To achieve various lattice orientations, the local coordinate frame of each gyroid unit cell was systematically redefined. In nTopology, the frame is defined by three mutually perpendicular axes ($\:x$, $\:y$, $\:z$), originating from a common reference point, set here at the global origin (0,0,0). The orientation of each gyroid model (G0–G5) was defined by adjusting the directions of the local $\:x$ and $\:y$-axes, while the $\:z$-axis direction is automatically established orthogonal to both $\:x$ and $\:y$ axes by the software. The specific vectors defining the frame axes for each gyroid model are summarized in Table 1. To quantify the orientation differences between models, the angle between the unit cell planes was determined. Each gyroid orientation can be described by the angle between the normal vectors of its defining planes. The general equation of a plane passing through a point $\:P=({x}_{0},{y}_{0},\:{z}_{0})\:$with a normal vector ($\:l,m,n$) is given by:

1 $$\:l\left(x-{x}_{0}\right)+m\left(y-{y}_{0}\right)+n\left(z-{z}_{0}\right)=0$$

To find the angle θ between two different orientations, the angle between their respective normal vectors was calculated using the following relationship:

2 $$\:\mathrm{cos}\theta\:=\frac{A.B}{\left|A\right|.\left|B\right|}$$

Where A and B are the normal vectors derived from the cross product of each model’s frame axes ($\:x$ and $\:y$ vectors)^27^.

**Table 1:** Frames of the unit cell planes.

Model	G0	G1	G2	G3	G4	G5
$\:x$. axis	(1,0,0)	(1,1,0)	(1,1,0)	(1,0,0)	(0,1,1)	(1,0,1)
$\:y$. axis	(0,1,0)	(0,0,1)	(0,1,1)	(0,1,1)	(1,1,0)	(0,1,0)

**Fig. 2:**
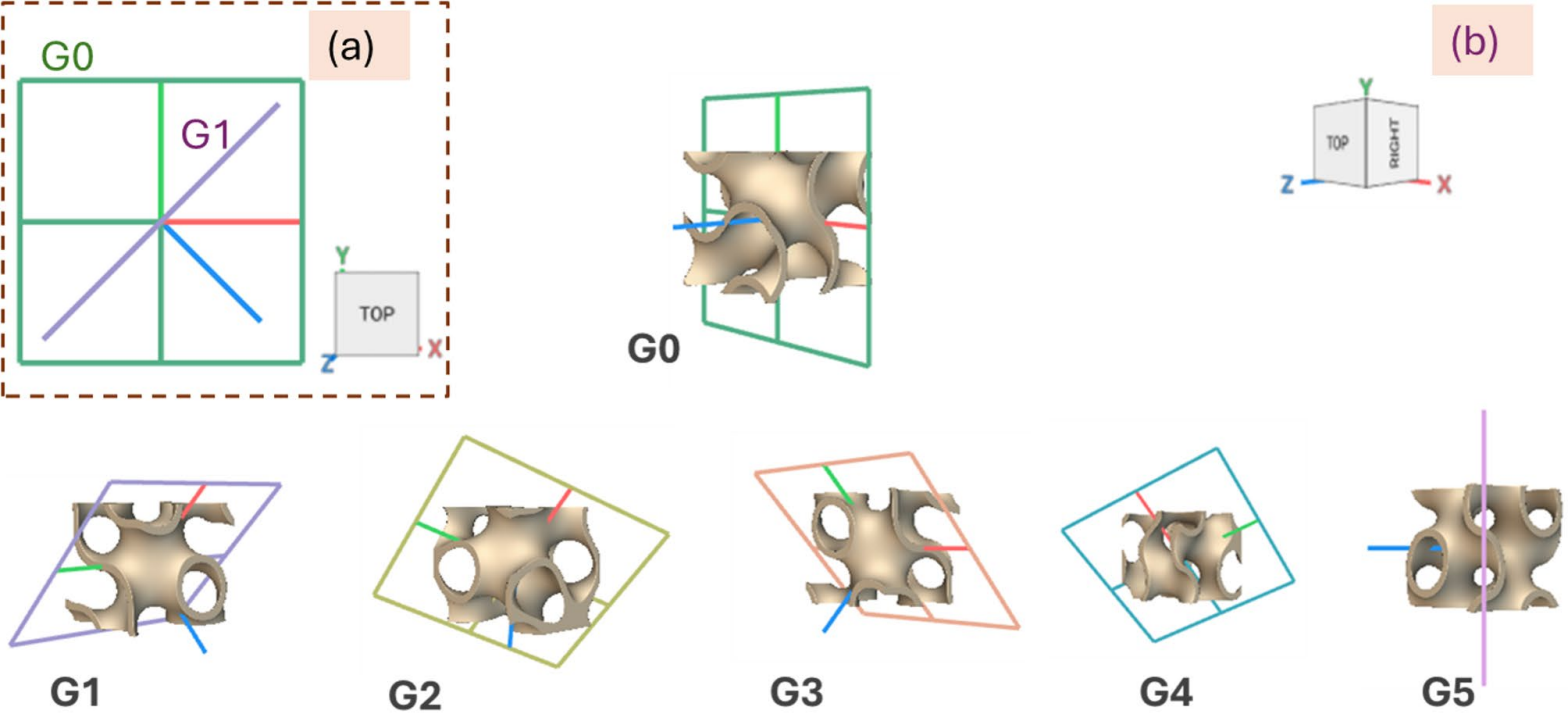
Different Gyroid unit cells with different orientations (**a**) Planes of G0 (green) and G1 (purple), (**b**) unit cells with different plane orientations.

Figure 2 illustrates the variations in unit cell orientation angles. In model G1, for example, the $\:x$-axis of the building frame is defined by the vector (1, 1, 0), while the $\:y$-axis is defined by (0, 0, 1). This configuration positions the $\:x$-axis at a 45° angle within the global $\:xy$-plane, and aligns the $\:y$ -axis vertically along the global $\:z$-direction (Fig. 2a). Consequently, the $\:z$-axis calculated as the cross product of the $\:x$ and $\:y$ vectors also lie at a 45° angle within the global $\:xy$-plane. Each model applies a unique combination of $\:x$- and $\:y$-axis vector directions, resulting in tailored orientation of the unit cell. These configurations include in-plane rotations (rotation occurs within a given global plane, e.g., G1, G2), out-of-plane rotations (a component that extends into another axis, e.g., G3, G5), and compound diagonal alignments across multiple global planes (e.g., G4), as shown in Fig. 2b. The angle between the unit cell build axis and the z-axis changed approximately from 0 to 30, 60, 90, 120°, and 180°, for models G0, G1, G2, G3, G4, and G5, respectively. The variation in building directions fundamentally alters the internal channel alignment and surface normal vectors, thereby enabling control over structural anisotropy, load path distribution, and energy absorption characteristics.

**Fig. 3:**
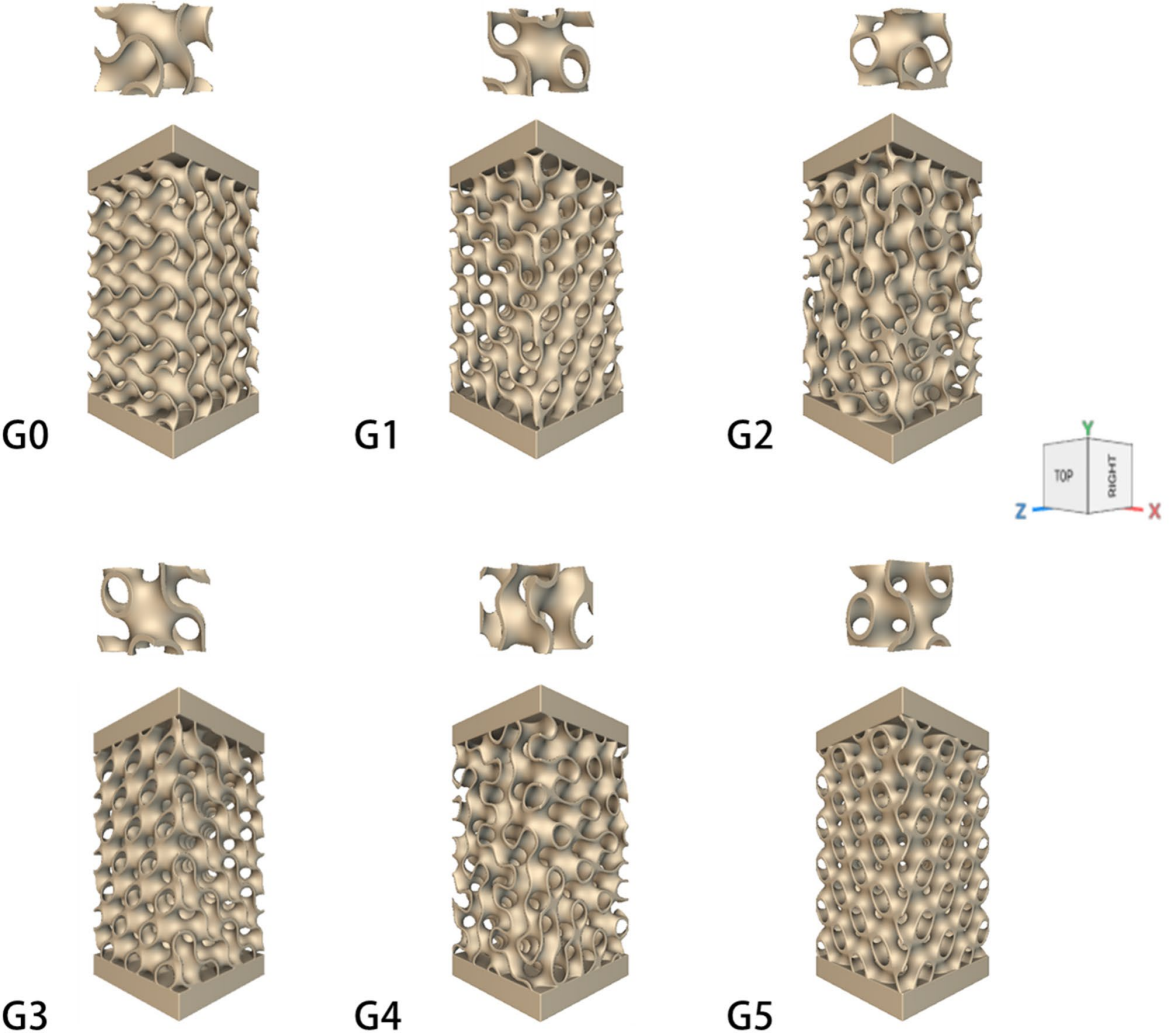
Designed compression test samples with different unit cell orientations.

A compression test specimen with dimensions of 12 × 12 × 20 mm (+ 2 mm solid top and bottom plate layers) is designed with a unit cell size of 4 × 4 × 4 mm and a wall thickness of 0.4 mm (Fig. 3). In addition to the original Gyroid structure, five designs of oriented gyroid structures were selected. The design structure was fabricated as 0.4 mm wall thickness and then expanded to 0.6 and 0.8 mm by numerical analysis.

The relative density (ρ^−^) of each model was calculated according to the porosity of each CAD model. The porosity, *P%*, was calculated according to the following relation:

3 $$\:P\:\%=100\frac{{V}_{void}}{{V}_{tot}}=100\left(1-\frac{{V}_{solid}}{{V}_{tot}}\right)$$

where V_tot_ is the volume of the total solid volume enclosing the unit cell, V_void_ is the void volume within the enclosing solid, and V_solid_ is the volume of the cells, obtained from the CAD models. The obtain porosity for each model is listed in Table 2.

**Table 2:** The calculated porosity for the proposed structure models with different wall thicknesses.

	Porosity %		
Wall thick. [mm]	0.4	0.6	0.8
G0	80.65	70.88	61.07
G1	80.45	70.62	60.73
G2	80.62	70.91	61.07
G3	80.44	70.61	60.70
G4	80.69	70.96	61.06
G5	80.28	70.43	60.50

### Fabrication of TPMS structures

**Fig. 4:**
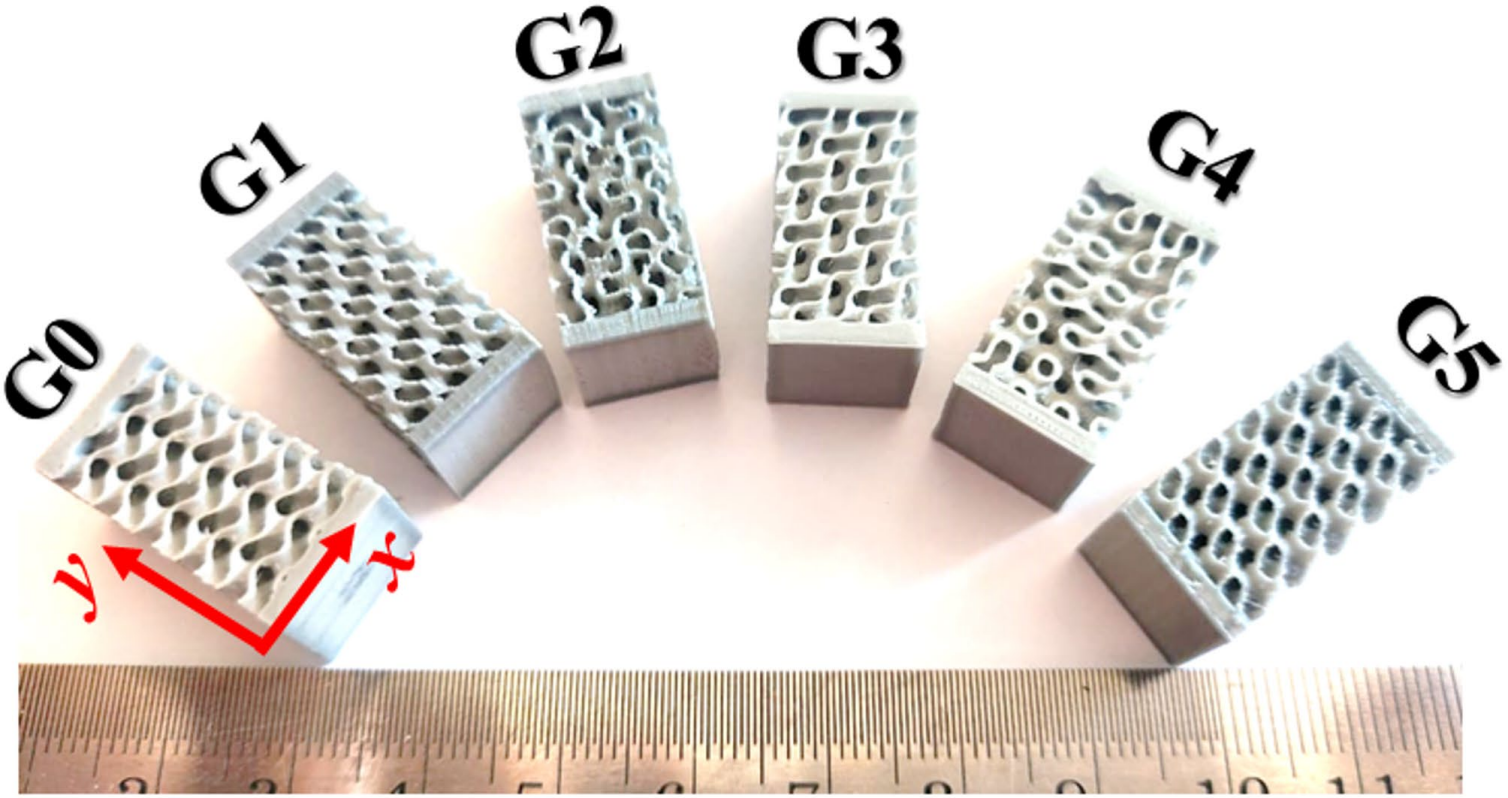
Fabricated PLA Gyroid samples printed using FFF technique.

The designs of gyroid structures have been exported from nTopology© program as .stl files. The printing process is performed using the FFF process by a Creality Ender-3 S1 Pro printer with a 0.2 mm nozzle for optimal results. All designed structures (original Gyroid and 5-oriented gyroid) are fabricated using eSUN™ PLA+. Figure 4 shows the printed gyroid specimens. The gyroid lattice geometries were developed following Design for Additive Manufacturing (DfAM) guidelines adapted to FFF processes, including minimum feature size ≥ 0.4 mm, self-support angles ≤ 45°, and layer-height-to-nozzle-diameter ratio ≤ 0.3. All models passed nTopology manufacturability checks and were experimentally validated: the printed specimens-maintained wall thickness within ± 0.03 mm of the CAD value, confirming dimensional fidelity and compliance with DfAM constraints. The printing parameters were set as follows: initial layer height of 0.16 while the remaining layer height set at 0.05 mm and a printing temperature of 205 °C, with the initial layer also printed at 220 °C temperature. The bed temperature was maintained at 65 °C. A gyroid infill pattern was utilized with a density of 100%. The print featured 5 outer walls and a print speed of 60 mm/s, while the initial layer was printed at 30 mm/s. The travel speed was set at 145 mm/s, with an initial layer travel speed of 50 mm/s. Printing acceleration was 450 mm/s², and travel acceleration was 1800 mm/s². Wall thickness was kept at 0.4 mm, with a retraction distance of 0.8 mm and a retraction speed of 45 mm/s. Finally, the flow rate was maintained at 98%. The main printing parameters are listed in Table 3.

**Table 3:** Printing parameters of the samples.

Nozzle temperature [°C]	Temperature of the bed [°C]	Infill density %	Printing speed [mm/s]
205	65	100	60

### Morphology and mechanical properties

The surface morphology and printing quality of the TPMS structural features were examined using scanning electron microscopy (SEM) with a TESCAN MIRA (Czech Republic). Prior to imaging, the specimens were coated with a thin layer of platinum to enhance conductivity. To evaluate the mechanical performance of the 3D-printed TPMS lattice structures (wall thickness: 0.4 mm), uniaxial compression tests were conducted in compliance with the ASTM D695-15 standard^28^. These tests were performed on a ZwickRoel testing system (GmbH & Co. KG, Ulm, Germany) equipped with a 10 kN load cell, applying a constant crosshead speed of 1 mm/min until a strain level of 50% was reached. A digital camera was employed to record the deformation during the compression testing, enabling detailed observation of the failure mechanisms. Each topology was tested using three identical samples. The load-displacement data recorded during testing were subsequently converted into nominal stress-strain curves for analysis.

Different approaches are reported in literature for estimating the effective cross-sectional area used in stress calculations. One approach treats the lattice as a solid structure, while another accounts for the internal geometry by averaging multiple cross-sectional measurements^22,29^. In this study, two stress calculation methods were employed: (1) the conventional engineering stress (σ), based on the external dimensions as a bulk solid, and (2) true stress (σ_t_), using an adjusted method considering the mean of the cross-sectional area measurements along the gyroid structure. For the latter, the cross-sectional area was determined through geometric analysis using nTopology’s integrated computational tools. Cross-sectional planes were systematically generated at 1 mm intervals along the compression loading axis, and the solid (non-void) area was calculated for each slice to establish the equivalent load-bearing cross-sectional area of the gyroid structure. The arithmetic mean of these discrete measurements was subsequently employed in true stress calculations.

The elastic modulus (E) was derived from the initial linear portion of the stress-strain curve using linear regression. The yield stress (σ_y_) was determined from the intersection of the stress-strain curve with a line offset by 0.2% strain from the linear region. The peak stress (σ_max_) was defined as the highest local stress achieved before a drop in stress levels. The specific value of this stress depends on the cell geometry and the relative density (ρ^−^) value before densification. The plateau stress (σ_pL_) was calculated as the average stress between 20% and 40% strain. Also, the first drop in the stress (after the peak) was estimated. Energy absorption per unit volume, W, was computed using the following relation:

4 $$W = 1/100\int\limits_{0}^{{\varepsilon 0}} {\sigma \:d\varepsilon }$$

where ε_0_ is the upper limit of the compressive strain in percentage, which is the compressive strain 50% ^30^.

### Numerical modeling

A finite element (FE) model was developed to predict the structural response and to investigate the collapse behavior and deformation mechanisms of various gyroid lattice structures under uniaxial quasi-static compression. The simulations were conducted using the commercial software Simulia^®^ 2024, ABAQUS/Explicit module. The choice of ABAQUS/Explicit was motivated by several factors: (1) the complex gyroid geometry with intricate curved surfaces and potential self-contact during large deformation compression, (2) the progressive failure mechanism involving element deletion and material degradation, which is more robustly handled in explicit formulations, and (3) the quasi-static nature of the compression process up to 50% strain, which involves significant geometric nonlinearity and contact interactions that can cause convergence issues in implicit solvers^31,32^. To ensure the validity of the explicit dynamic solution for quasi-static conditions, the energy balance was carefully monitored throughout the analysis, and no mass scaling was applied. The kinetic energy was maintained below 5% of the internal energy throughout the deformation process, confirming that inertial effects were negligible, and the solution remained quasi-static in nature.

**Fig. 5:**
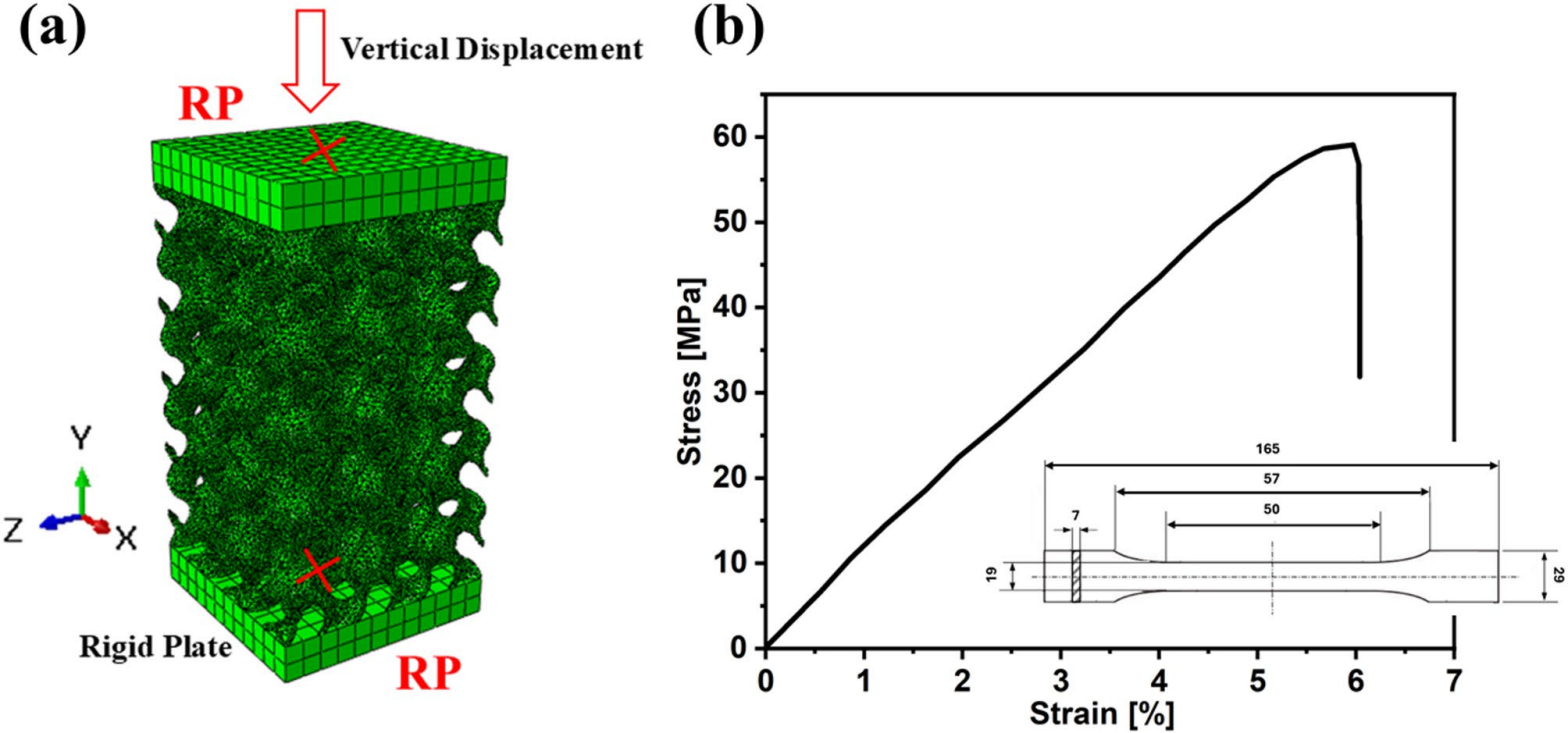
(**a**) FE model of gyroid lattice under uniaxial compression with rigid plates; vertical displacement applied via the top reference point (RP), bottom RP fully fixed, (**b**) Stress–strain curve of a 3D-printed dog-bone sample (PLA), the sample geometry is shown in the inset.

The 3D gyroid geometries were generated and initially meshed using nTopology and exported to ABAQUS in a compatible format. For accurate results, tetrahedral elements with a mesh size of 0.2 mm were used, providing a balanced trade-off between capturing the complex curvature of the gyroid surfaces, ensuring agreement with experimental data, and minimizing computational cost. The modeled structures originally had a wall thickness of 0.4 mm, which was systematically increased to 0.6 mm and 0.8 mm, while maintaining the same lattice configuration along the three global axes. Details of the numerical model setup are illustrated in Fig. 5a. The FEA model was created by placing the lattice structure between two rigidly designed plates. The bottom plate was fully constrained (all degrees of freedom restricted), while the upper plate was allowed to have a vertical displacement only, compressing the structure up to 50% of its initial height. A reference point was assigned to the upper plate to record the applied displacement and corresponding reaction force, which were subsequently used to generate engineering stress-strain curves.

The gyroid structures were meshed using ten-node modified quadratic tetrahedral elements (C3D10M), with the total element count ranging between 210,000 and 355,000. General contact conditions were applied throughout the model to ensure load transfer between the plates and lattice and to account for general-contact between lattice walls. The contact interaction was defined using “hard” normal behavior and a tangential “penalty” formulation with a friction coefficient of 0.3 ^33^.

The material behavior was modeled as an elastic-plastic material model, based on experimental tensile test data of standard PLA specimens. The assigned material properties for PLA were: Young’s modulus = 1031 MPa, Poisson’s ratio = 0.34, and yield strength = 30.1 MPa, which are consistent with values reported previously^34,35^. The plastic behavior of PLA was defined by converting the tensile test engineering stress–strain curve (Fig. 5b), measured from printed dog-bone samples to true stress–plastic strain. This data was used directly in the FEA material mode. Damage initiation was modeled using the ductile damage criterion. A linear damage evolution law and failure at which the material loses its load-carrying capacity was based on displacement at failure and was set to 0.05 mm. Element deletion was enabled to simulate material removal upon failure during the deformation process and prevent excessive deformation that may terminate the analysis.

## Results and discussion

### Morphology of as printed gyroid model

**Fig. 6:**
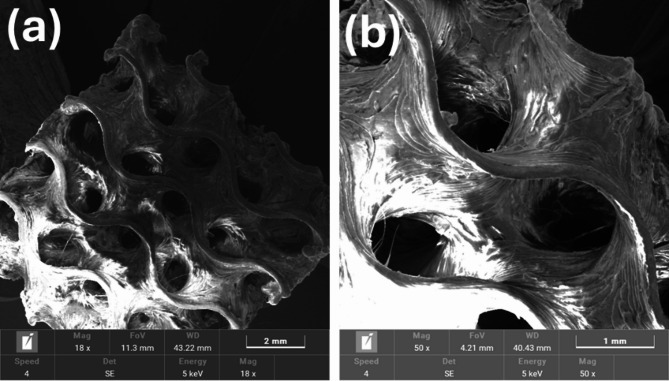
(**a**) SEM micrograph of the gyroid design, (**b**) SEM close-up of the openings of the gyroid and strut.

The SEM image of the as-printed G0 model is shown in Fig. 6. It confirms the successful fabrication of the gyroid lattice, accurately replicating the designed periodic porous architecture. In Fig. 6a, the G0 sample exhibits a well-preserved gyroid geometry with continuous struts and interconnected pores, reflecting the high fidelity of the printing process at the macroscale. Figure 6b reveals surface-level imperfections, including rough textures and partially fused filament layers, which are commonly associated with limitations characteristic of FFF technology^36^.

These defects are primarily attributed to factors such as infill overlap, limited nozzle resolution, and insufficient bonding between adjacent layers. Despite these challenges, the structure maintains overall geometric integrity. Minor variations in wall thickness, ranging from 0.393 mm to 0.421 mm, are likely due to material shrinkage and localized overlap during filament deposition. Such deviations influence the relative density and are linked to the interplay between design parameters, like unit cell size and wall thickness, and the resolution constraints of the 3D printing system^37^. Although potential improvements, such as using finer nozzles or adjusting the unit cell design, could minimize these discrepancies, such process optimization lies beyond the scope of this study.

### Homogenization analysis

**Fig. 7:**
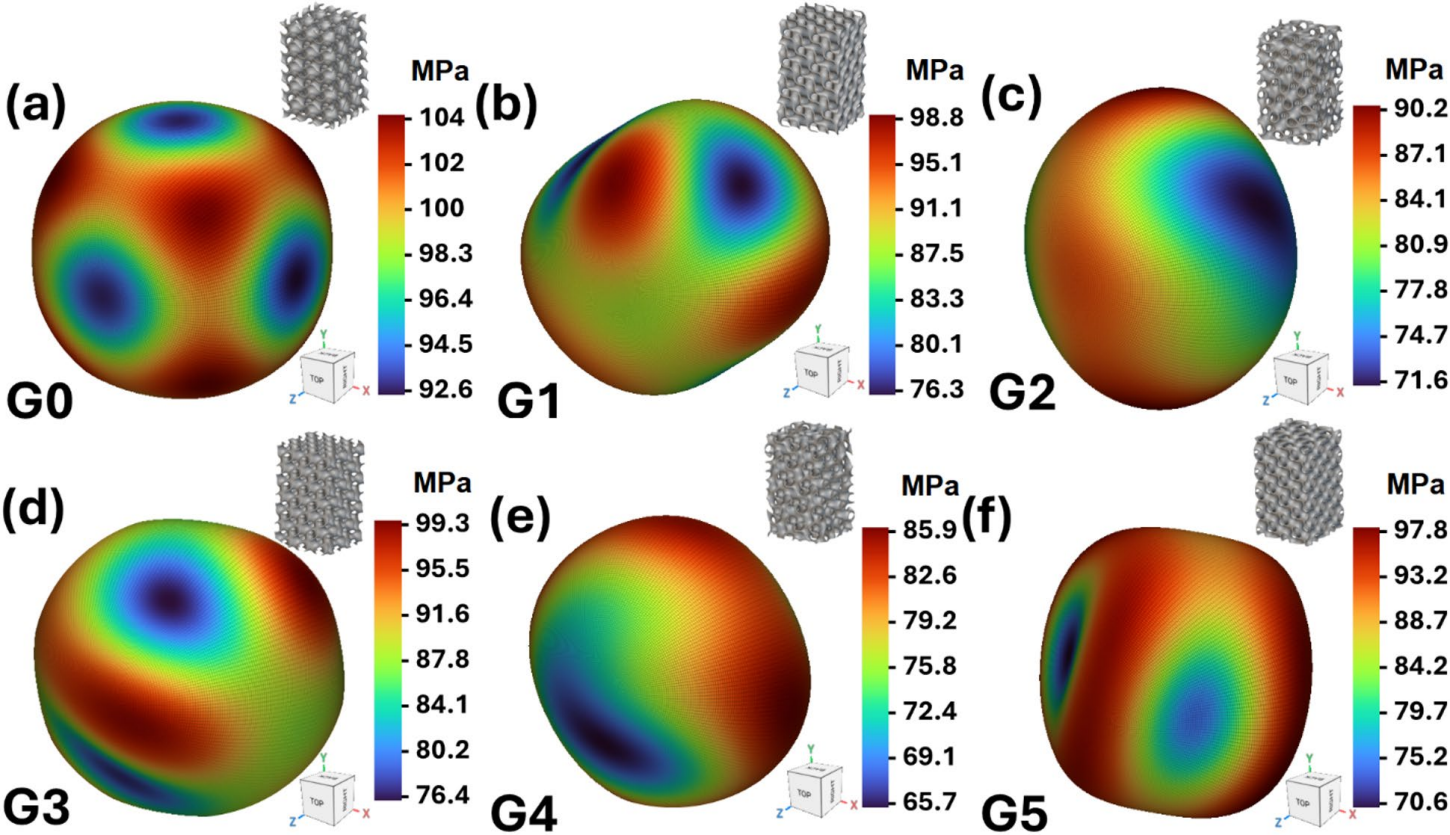
Homogenized relative elastic modulus for the proposed Gyroid models: (**a**) G0; (**b**) G1; (**c**) G2; (**d**) G3; (**e**) G4; (**f**) G5.

It is essential to quantify and visualize the degree of anisotropy of TPMS structures to optimize their mechanical performance across different application scenarios. Particularly in energy absorption and load-bearing applications, isotropic mechanical behavior ensures predictable and uniform response under multiaxial loading conditions, which is critical in applications such as medical implants, automotive, and aerospace safety structures^38^. A methodology to characterize anisotropy involves calculating the normalized elastic modulus E/E_max_ in all spatial directions, where E is the local elastic modulus and E_max_ is the maximum across all directions. For a perfectly isotropic material, the modulus surface is spherical (E = E_max_) with a Zener ratio of 1, indicating uniform properties^39^. In this study, these surfaces were generated using directional homogenization via nTopology software and are visualized in Fig. 7(a-f).

**Fig. 8:**
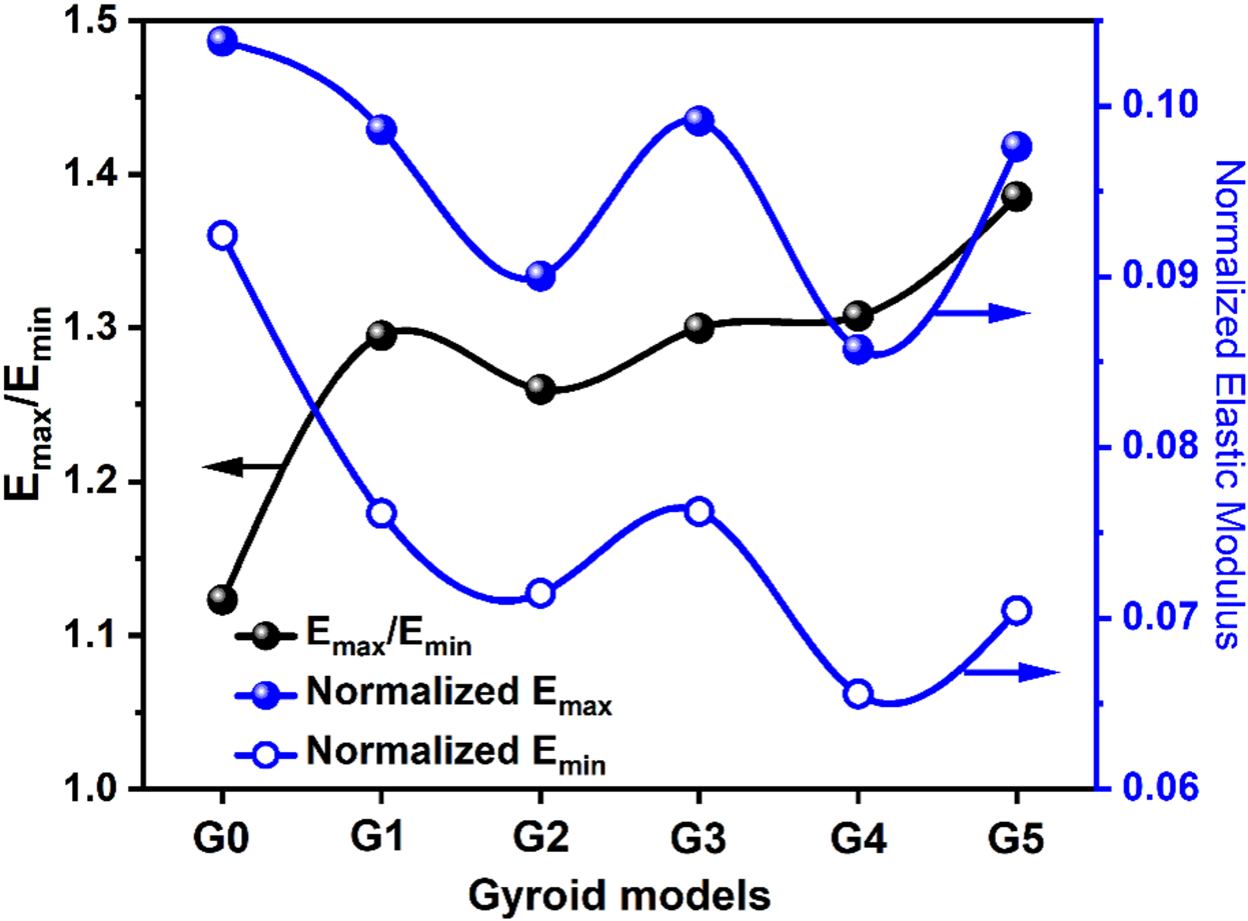
The ratio of the maximum to minimum elastic modulus and the normalized elastic modulus for different Gyroid models.

As shown in Fig. 7a, model G0 displays the most isotropic response, evidenced by the nearly spherical Young’s modulus surface, indicating uniform mechanical properties in all directions. This isotropy is advantageous for applications involving random or multi-axial loading. Models G1, G2, G3, and G4 show a gradual deviation from isotropy, with the Young’s modulus distributions indicating preferential stiffening along specific axes, as shown in Fig. 7(b-e). This behavior aligns with known results where structural orientation relative to loading affects stiffness anisotropy^40^. Model G5 exhibits pronounced anisotropic behavior, Fig. 7f, where the modulus is significantly higher along the vertical z-axis, consistent with the strut orientation observed in the unit cell geometry. Such behavior is beneficial when directional stiffness is desired, but may compromise energy absorption under off-axis loads^41^.

Figure 8 presents a quantitative analysis of anisotropy variation across all gyroid orientations through three key metrics: the anisotropy ratio (E_max_/E_min_), normalized maximum elastic modulus (E_max_), and normalized minimum elastic modulus (E_min_). The anisotropy ratio reveals that G0 maintains the most isotropic behavior with a ratio of 1.12, while G5 exhibits the highest anisotropy at 1.37. Remarkably, the orientations (G1-G4) show relatively similar anisotropy levels ranging from 1.25 to 1.30, suggesting a plateau effect in directional dependence on rotation angles. The normalized maximum modulus demonstrates an individual pattern, starting high at G0 (0.105), decreasing to a minimum at G2 (0.072), then recovering toward G5 (0.071). Conversely, the normalized minimum modulus shows an inverse trend, beginning at G0, reaching a local minimum at G4, and partially recovering at G5. This matching behavior indicates that gyroid orientations create trade-offs between maximum stiffness in preferred directions and minimum compliance in weak directions^42^.

From a structural design perspective, G0 would be preferred in isotropic energy absorption systems (e.g., crash absorbers), whereas G5 could be suitable for applications requiring stiffness reinforcement along a primary axis, such as orthopedic implants or aerospace rib structures. Despite the apparent geometric complexity and lack of intuitive symmetry, most gyroid-based TPMS structures approximate cubic symmetry, although the anisotropic modulus surfaces reveal subtle variations.

### Experimental and FE validation

**Fig. 9:**
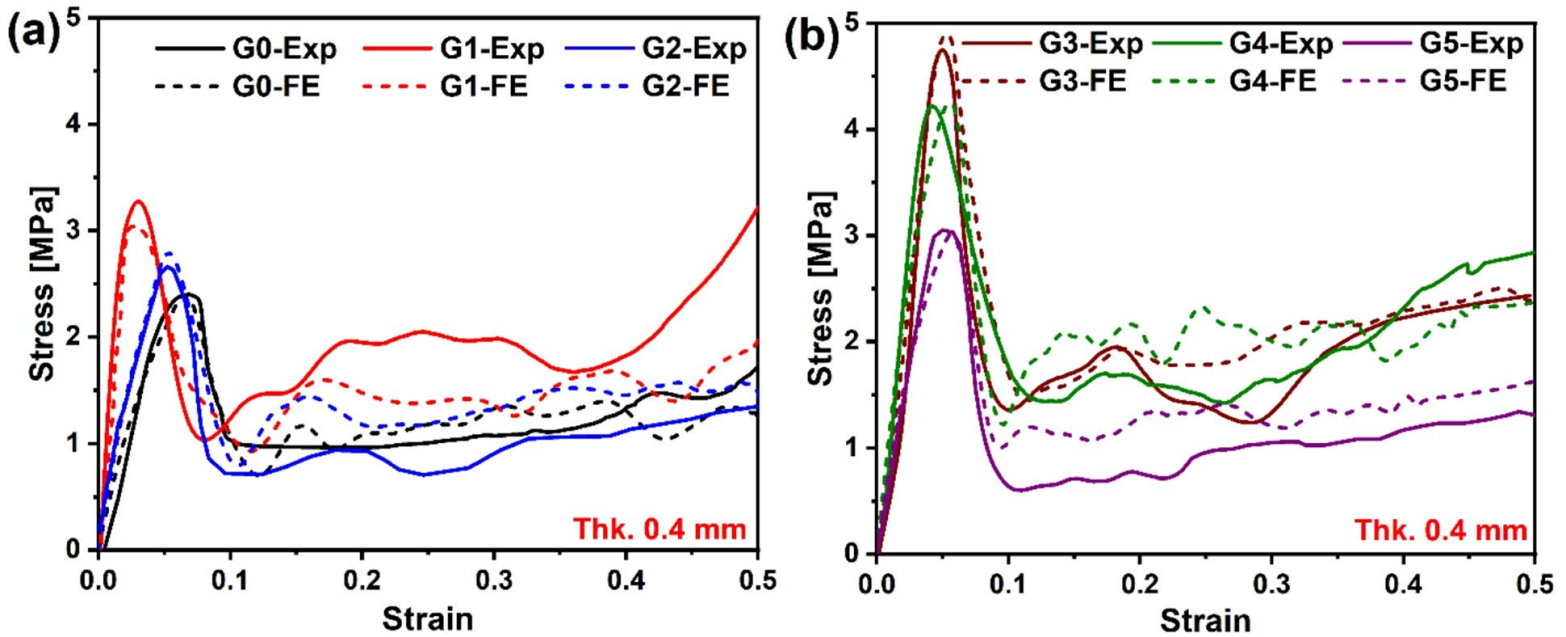
Stress-strain characteristics obtained from the experiment and FE for 0.4 mm thickness models (**a**) G0, G1, and G2, (**b**) G3, G4, and G5.

A comparative analysis of the stresses obtained from FE and experimental (Exp) data was conducted for six 3D-printed TPMS lattice structures (G0-G5) with a wall thickness of 0.4 mm under uniaxial quasi-static compression, as illustrated in Fig. 9. The results demonstrate a strong correlation between the FE predictions and experimental measurements, affirming the validity of the modeling assumptions. Particularly, the initial stress drop observed in the experimental stress-strain curves aligns closely with the FE results, indicating that the simulated boundary conditions, material model, and meshing accurately replicate the physical testing scenario.

The deviation between the response derived from the FE and the corresponding average experimental values ranged from 0.04% to 20%. The most significant discrepancies were observed in the plateau stress predictions across all models. This overestimation is particularly evident in the plastic region, a phenomenon frequently reported in literature and attributed to the omission of fabrication-induced imperfections in FE models^43^. These imperfections are known to reduce the critical buckling loads in structures under compression. As highlighted by Gonzalez and Nuno, neglecting manufacturing irregularities can result in an overestimation of the quasi-elastic stiffness of lattice structures by as much as a factor of 12 compared to experimental observations^44,45^ .

### Mechanical properties

**Fig. 10:**
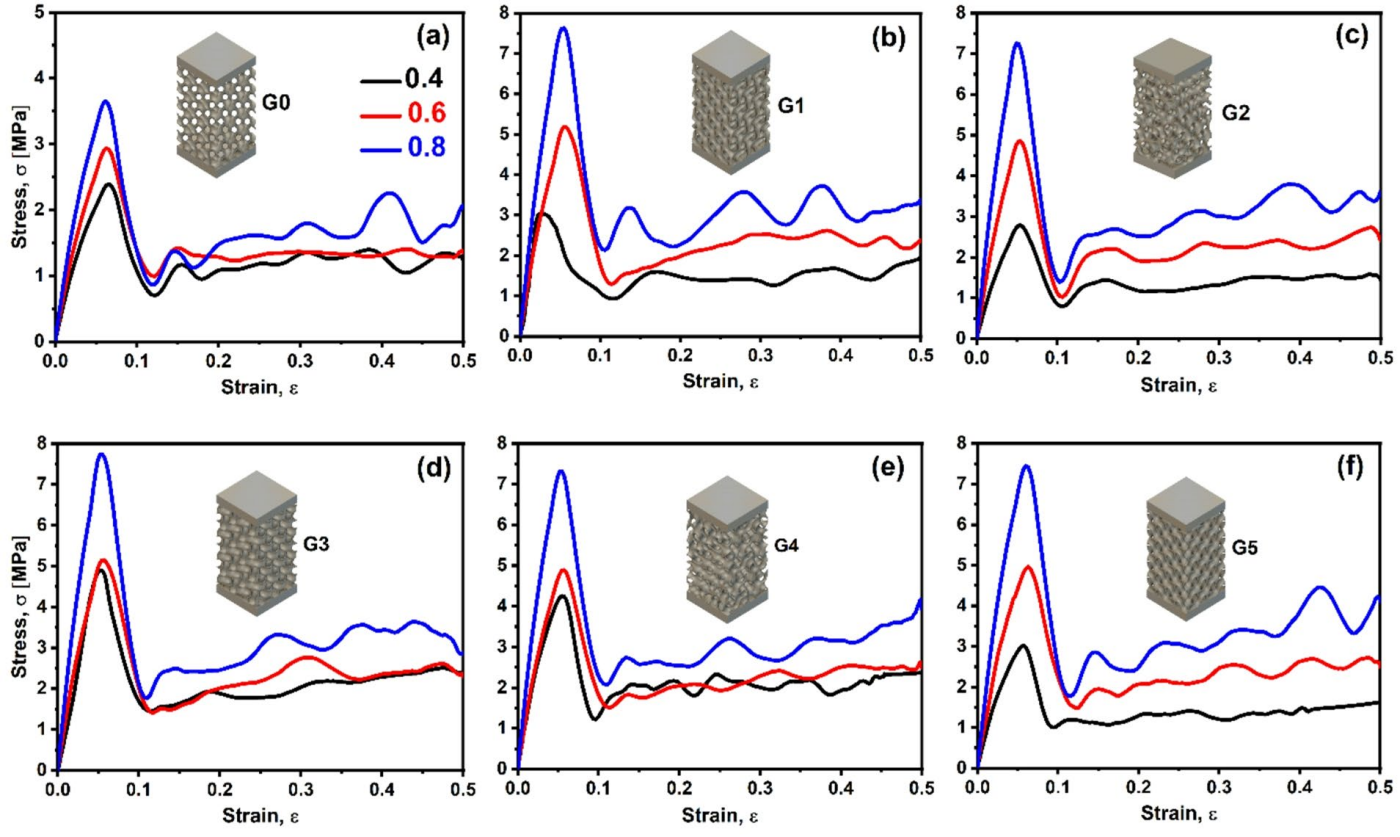
Numerical compressive stress-strain curves of G- TPMS structures with different wall thickness, G0–G5, (**a**–**f**).

The compression test results for the gyroid lattice structures with varying wall thicknesses, as depicted in Fig. 10 (a-f), reveal distinct stress-strain behaviors that reflect the influence of structural geometry on mechanical performance. Despite the symmetric boundary conditions applied in the FE model, some stress contours in Fig. 10 exhibit apparent asymmetric patterns. This asymmetry arises from several factors: (1) the inherent geometric complexity of the gyroid TPMS structure creates non-uniform load paths even under symmetric loading conditions, (2) the progressive failure mechanism involves localized buckling and strain localization that naturally develops along paths of least resistance, which may not align with the geometric centerline, and (3) the element deletion feature enabled in the damage model removes failed elements asymmetrically as plastic deformation progresses, contributing to the observed stress redistribution patterns^46,47^. Additionally, the different orientations of the gyroid unit cells (G1-G5) create varying internal architectures that fundamentally alter stress flow patterns, making perfect symmetry unlikely despite symmetric boundary conditions.

For all configurations, the stress-strain curves exhibit three principal stages: an initial linear-elastic region, a peak stress marking the onset of structural yielding, and a post-yield plateau dominated by progressive cell wall collapse and densification. The densification stage is not evident because the structure was not compressed up to such a level of strain^48^. The magnitude of the peak stress and the nature of the post-yield response are both strongly influenced by wall thickness, reflecting the dependency of mechanical strength on the relative density (ρ^−^), in agreement with the classical scaling laws proposed by Gibson and Ashby^49^. For model G0 (Fig. 10a), the stress-strain curve exhibits a pronounced initial peak stress ( from 2.5 to 3.8 MPa for 0.4 and 0.8 mm thickness), followed by a sharp decline and a subsequent plateau with oscillatory stress values around 1–2 MPa up to 0.5 strain. This behavior suggests a bending-dominated deformation mechanism, where such walled structure undergoes elastic buckling followed by progressive cell wall collapse, consistent with observations in low-density cellular solids^33,50^. The oscillatory plateau indicates energy dissipation through localized bending and folding of the lattice struts.

As wall thickness increases, the initial peak stress increases up to 7.79 MPa for G3-0.8 thickness, for example (Fig. 10d), with a gradual post-peak softening and a sustained plateau increase. The responses of G1, G3, and G4 show undulating stress patterns post-yield, while G2 and G5 exhibit a more pronounced upward trend, particularly beyond a strain of 0.3 (Fig. 10c, f). This transition suggests a shift toward a mixed deformation mode, combining bending and stretching, driven by the increased wall thickness, enhancing strut rigidity. The stretching component becomes more dominant as thicker walls resist buckling, allowing load transfer through axial deformation, aligning with findings in thicker gyroid structures where stretching contributions increase with relative density^51,52^.

The initial peak corresponds to the elastic limit, where the lattice deforms uniformly, followed by a stress drop due to the onset of localized instability (e.g., strut bending or shear band formation). The plateau region reflects progressive crushing, with the amplitude of oscillations decreasing as wall thickness increases, indicating reduced energy dissipation through bending. For G4 and G5, the rising stress at higher strains suggests densification, where cell walls begin to contact and bear load compressively, a behavior typical of cellular materials approaching full compaction^53,54^. The physical reason behind this behavior lies in the interplay of geometry and material properties: thinner walls favor bending due to lower bending stiffness, while thicker walls enhance axial stiffness, promoting a mixed or stretching-dominated response. These observations highlight the tunability of gyroid lattices for energy absorption applications, with wall thickness serving as a key design parameter to tailor deformation mechanisms.

**Fig. 11:**
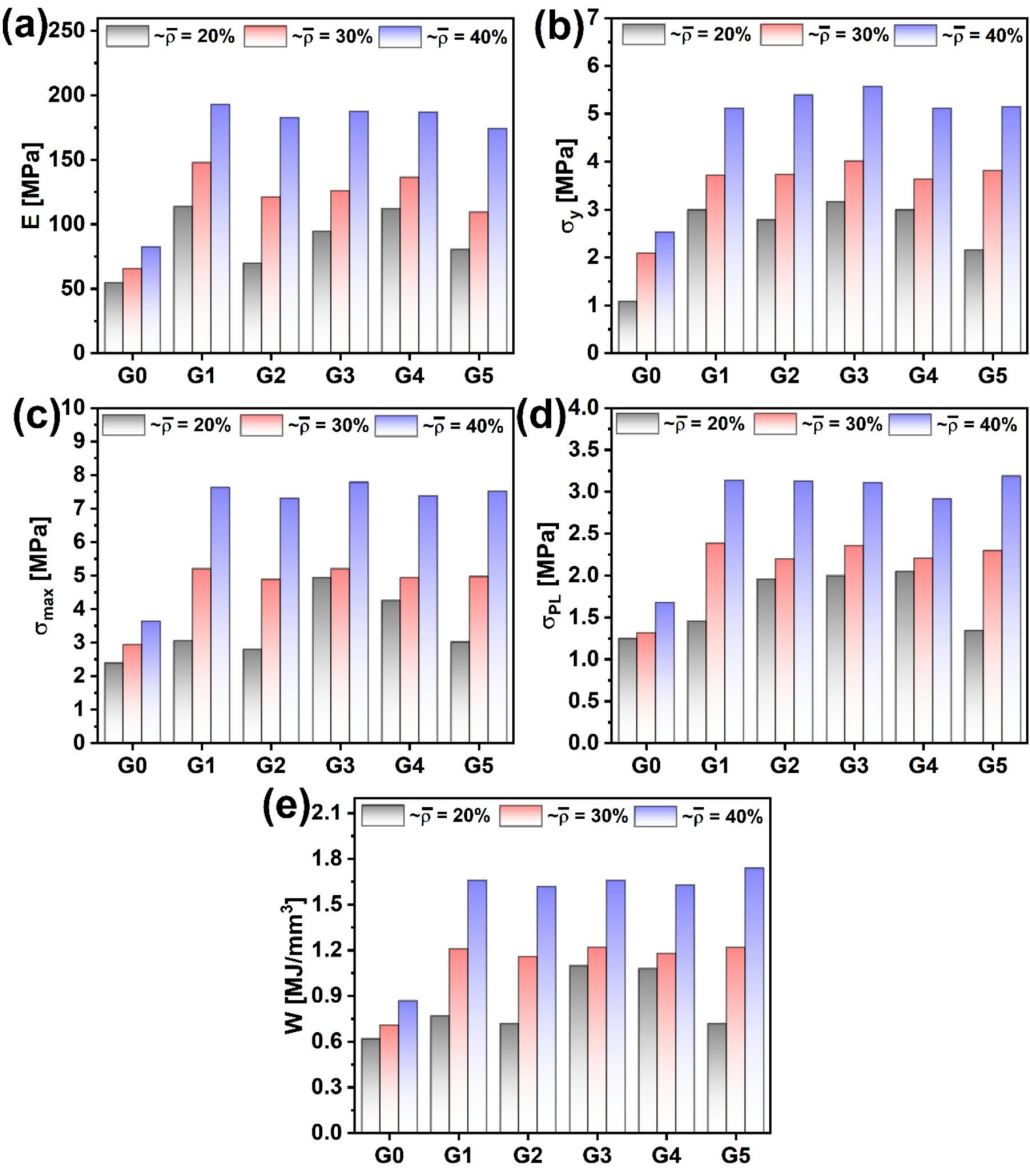
Numerical mechanical properties of proposed TPMS lattices: (**a**) Young’s modulus, (**b**) Yield stress, (**c**) peak stress, (**d**) plateau strength, and (**e**) absorbed energy.

The mechanical characterization of the gyroid structures G0 through G5 under compressive loading was further supported by quantitative analysis of stiffness, strength, and energy absorption as a function of porosity, shown in Fig. 11 (a) through (e). As expected from cellular solid theory, a reduction in porosity (i.e., increasing in relative density and wall thickness) led to systematic enhancement in mechanical performance across all structures. For instance, Young’s modulus (E), shown in Fig. 11a, increased substantially as relative density increased from 20% to 40%. At ~ 40% ρ־, G1 exhibited the highest stiffness (193 MPa), followed closely by G3 and G4 (both ∼187 MPa), increased by 134% and 125% over G0, respectively. The enhanced stiffness in G1 and G3 can be attributed to their relatively aligned and interconnected strut networks (Fig. 3), enabling effective axial load transfer, a treat of stretch-dominated architectures. In contrast, the lower stiffness of G0 suggests a bending-dominated regime, where deformation occurs primarily via flexural modes of slender struts with limited load path continuity.

Similarly, yield stress (σ_y_), as illustrated in Fig. 11b, followed the same hierarchical trend. At 40% ρ־, G1 and G3 reached yield stresses above 5 MPa, while G0 remained below 3 MPa. This confirms the superior load-bearing initiation in topologies with structured strut alignment, where elastic instabilities are delayed due to geometric redundancy and localized confinement. Similar observations demonstrated that architected lattice materials with controlled alignment and periodicity exhibit enhanced initial stiffness and delayed onset of buckling due to structural redundancy and distribution of localized stress fields^55^. Likewise, Maskery et al. emphasized that well-aligned TPMS topologies, such as the gyroid, show greater resistance to elastic instability because of their continuous and triply periodic architecture, which promotes uniform load transfer and geometric confinement under compression^56^. Furthermore, Babaee et al. revealed that lattices with orientational symmetry and nodal connectivity exhibit strain-stiffening behavior that suppresses premature localized collapse^57^. The trend for maximum compressive strength (σ_max_), plotted in Fig. 11c, further emphasizes the dominant role of topology under quasi-static compression. An enhancement over G0 by 105, 77, and 114% by he G3 model, as the thickness increases by 0.4, 0.6, and 0.8 mm, respectively.

The plateau stress (σ_PL_), shown in Fig. 11d, which reflects the sustained stress after yield during progressive collapse, reveals a more nuanced difference between the geometries. At 60% porosity, the σ_PL_ values for the oriented structures were markedly higher than for G0, and the trends vary depending on the constituent topology, suggesting improved energy absorption and structural resilience. This is particularly critical for applications such as impact protection, where load must be dissipated over time without brittle failure. Most remarkably, the absorbed energy density (W), integrated over strain and shown in Fig. 11e, presents a holistic view of performance by combining both strength and deformability. There is a clear modification in energy absorption per unit volume at ~ 20% ρ־, except for the G0 model. However, at both 40% and 30%, the values remain comparable. At higher porosity levels, the G1, G2, and G5 gyroid structures exhibited comparable energy absorption capacities; this trend closely follows their respective maximum peak stress behavior. The alignment between energy absorption and peak stress behavior implies that the dominant deformation mechanisms at high porosity are governed more by overall structural topology than by subtle geometrical differences, resulting in comparable stress distribution and failure modes across these designs. At a higher relative density (40%), G5 emerged as the best energy absorber (1.8 MJ/mm³), followed closely by G3 and G4. This is in direct alignment with the post-yield behavior observed in the corresponding stress-strain curves. The sustained load-bearing and progressive collapse in these models indicate a quasi-ductile failure mechanism, facilitated by interconnected gyroid strut networks after orientation.

Across all metrics, the coupling of geometric design and relative density plays a crucial role in dictating deformation mechanisms. Stretch-dominated topologies (e.g., G1, G3, G5) benefit from structural alignment, high nodal connectivity, and axial stiffness, resulting in enhanced stiffness, strength, and energy absorption. In contrast, G0 and G2, with less favorable geometrical coherence, predominantly deform via bending, yielding lower mechanical performance despite similar porosities. These findings align well with the framework of architected materials design, where optimization of mechanical behavior is achieved not merely by increasing density but by judiciously tailoring internal geometry^58^. The strong correlation between experimental trends and theoretical predictions reinforces the effectiveness of TPMS-based structures, particularly gyroid families, in advanced lightweight structural applications.

**Fig. 12:**
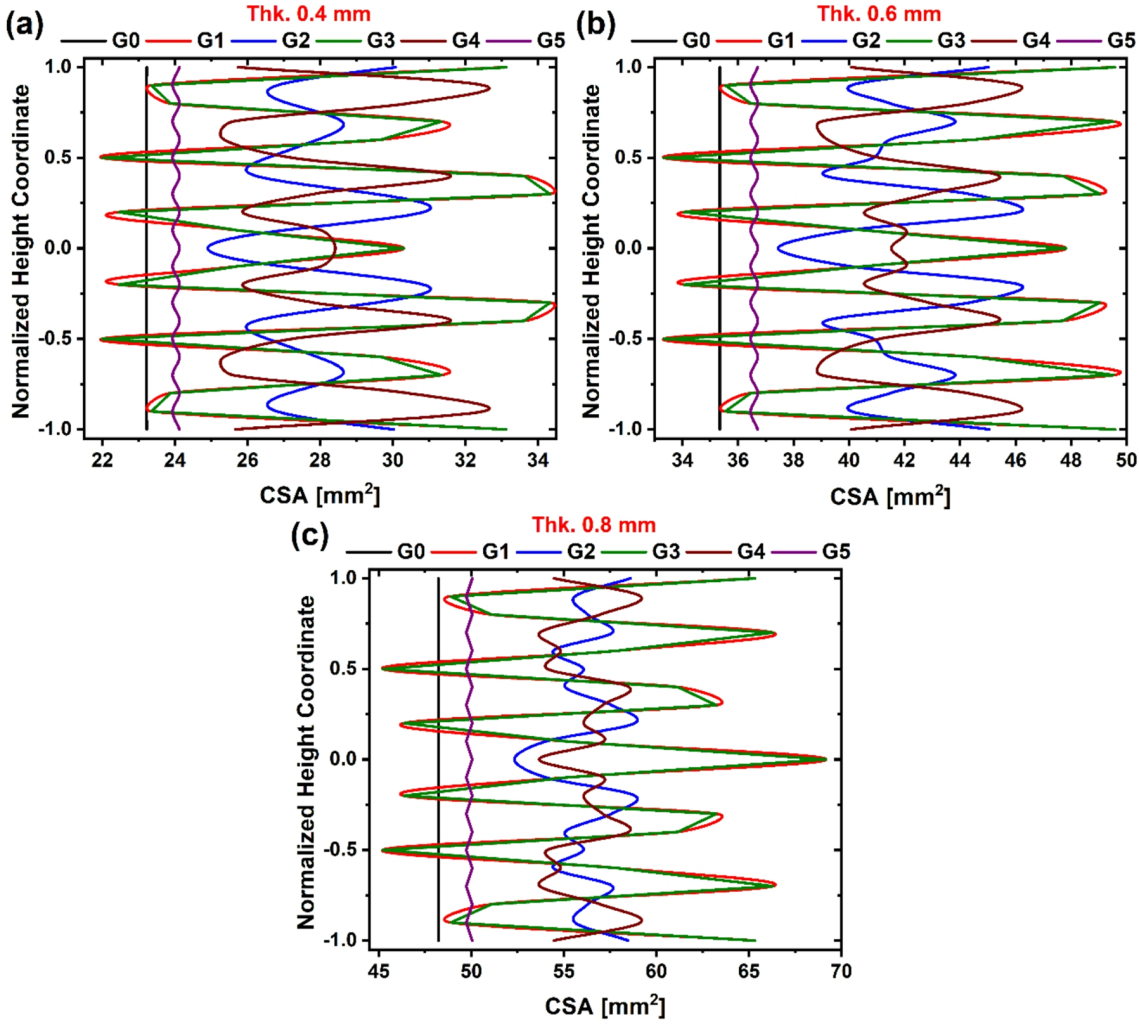
Cross-sectional area variation of the topology along the height with different wall thickness, (**a**) 0.4 mm, (**b**) 0.6 mm and (**c**) 0.8 mm.

The stress-strain calculations were repeated using the actual cross-sectional area (CSA) extracted from the CAD models, sliced at 1 mm intervals along the height. Figure 12 (a-c) illustrate the variation in CSA along the normalized height coordinate for all studied TPMS lattice topologies (G0-G5). The normalized height axis is defined such that zero corresponds to the mid-height of the structure. The CSA values exclude contributions of the top and bottom solid plates. All TPMS configurations exhibit non-uniform CSA distributions along their vertical axis, except for G0 and G5, which maintain nearly constant CSA profiles throughout their height. These two topologies consistently exhibit the lowest CSA values across all thicknesses, indicating lower structural mass density and a more uniform material distribution along the building direction.

In contrast, models G1 and G3 exhibit similar CSA profiles characterized by the highest peak values among all topologies-approximately 34.3, 49.5, and 69.2 mm² at wall thicknesses of 0.4, 0.6, and 0.8 mm, respectively. These values reflect localized regions of material accumulation, which may correspond to increased stiffness and load-bearing capacity. Meanwhile, G2 and G4 demonstrate more pronounced oscillations in their CSA profiles, suggesting higher geometric periodicity, which may lead to localized stress concentrations or deformation instabilities under loading. Despite these geometric differences, the average CSA values within each thickness group remain relatively close. For instance, at 0.4 mm wall thickness, average CSA values range between ~ 23 and ~ 28 mm². This narrow variation supports the use of average CSA for stress calculations, ensuring consistency across topologies and improving the reliability of comparative stress-strain analysis^59,60^.

**Fig. 13:**
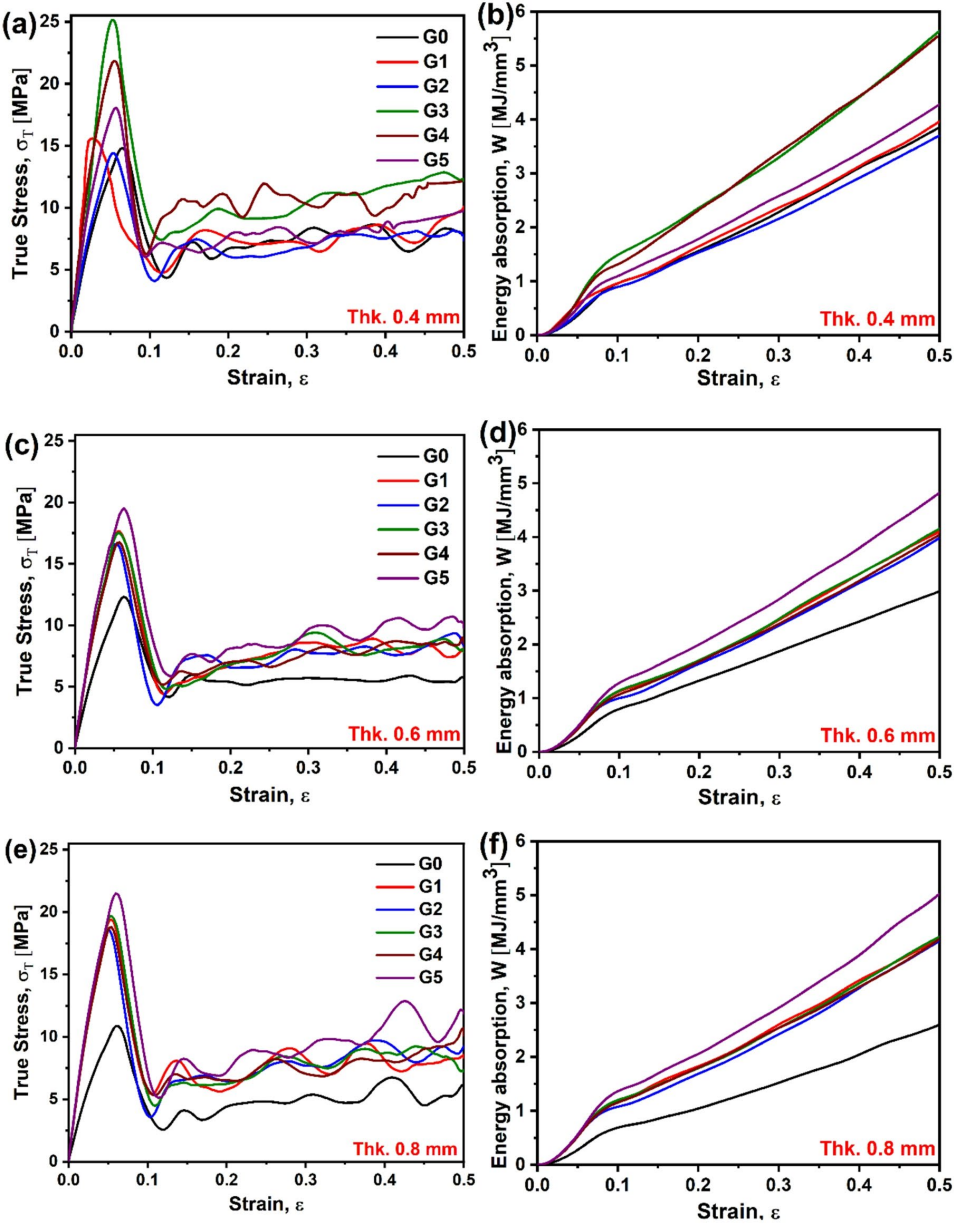
Mechanical compression properties and absorbed energy for G0 to G5 lattice models, calculated using the as-printed cross-sectional area, for wall thickness (**a**, **b**) 0.4 mm, (**c**, **d**) 0.6 mm, and (**e**, **f**) 0.8 mm.

Figure 13 illustrates the true stress-strain behavior and corresponding energy absorption for TPMS-based lattice structures G0 to G5, evaluated using the actual as-printed cross-sectional area (CSA). This methodology enables a more accurate reflection of mechanical performance than nominal area calculations, which often overestimate stress by ignoring geometric variation along the specimen height. At 0.4 mm wall thickness (Fig. 13a), G3 records the highest peak stress at 25.2 MPa, followed by G4 at 21.9 MPa. Meanwhile, G1 and G2 exhibit lower peak values, indicating moderate stiffness. The corresponding energy absorption ranks G3 (5.64 MJ/mm³) highest, highlighting the role of structural confinement and load path distribution, as shown in Fig. 13b.

At 0.6 mm thickness (Fig. 13c, d), G5 overtakes all others in both peak stress (19.8 MPa) and energy absorption (4.8 MJ/mm³), suggesting that its near-uniform CSA becomes more mechanically effective at higher relative densities. This marks a 43% increase in stress and a 63% improvement in energy absorption compared to G0. G1-G4 maintain intermediate values, with less pronounced differences between them, indicating convergence of performance at this intermediate density range. At 0.8 mm thickness (Fig. 13e, f), G5 continues to dominate, achieving a peak stress of 21.7 MPa and energy absorption of 5 MJ/mm³, while G0 remains the weakest performer. The data confirms that the mechanical efficiency of G5 scales significantly with thickness, likely due to improved connectivity and a transition from bending- to stretch-dominated behavior.

When compared with previously discussed engineering stress-strain results (Fig. 10), the current analysis reveals that at lower ρ^−^ levels (~ 80% porosity, 0.4 mm thickness), localized material distribution or the nominal one didn’t affect the mechanical properties significantly. G3 recorded the peak stress level, while G4 showed the maximum plateau stress in both cases. In contrast, with increasing ρ^−^, G5 demonstrated a favorable trade-off between stiffness and energy absorption-an essential characteristic for applications requiring crashworthiness and structural integrity, as supported by the TPMS design. This is attributed to the uniform material distribution (present as CSA in this study) (Fig. 12). This geometry-dependent difference can be explained by the internal material distribution, which affects the failure behavior and load-bearing capacity of the structure^61^. Specifically, Zhao et al. demonstrated that the progression of stress during the compression of TPMS structures is strongly influenced by how material is distributed along the loading axis^62^. Furthermore, Downing et al. enhanced the mechanical performance of TPMS lattices by strategically modifying the spatial distribution of material within the structure^63^. Therefore, internal material distribution is an important factor that affects the mechanical properties of TPMS structures; however, beyond lightweight structures (as in 0.4 mm in this study), it is not necessary.

### Deformation behavior

**Fig. 14:**
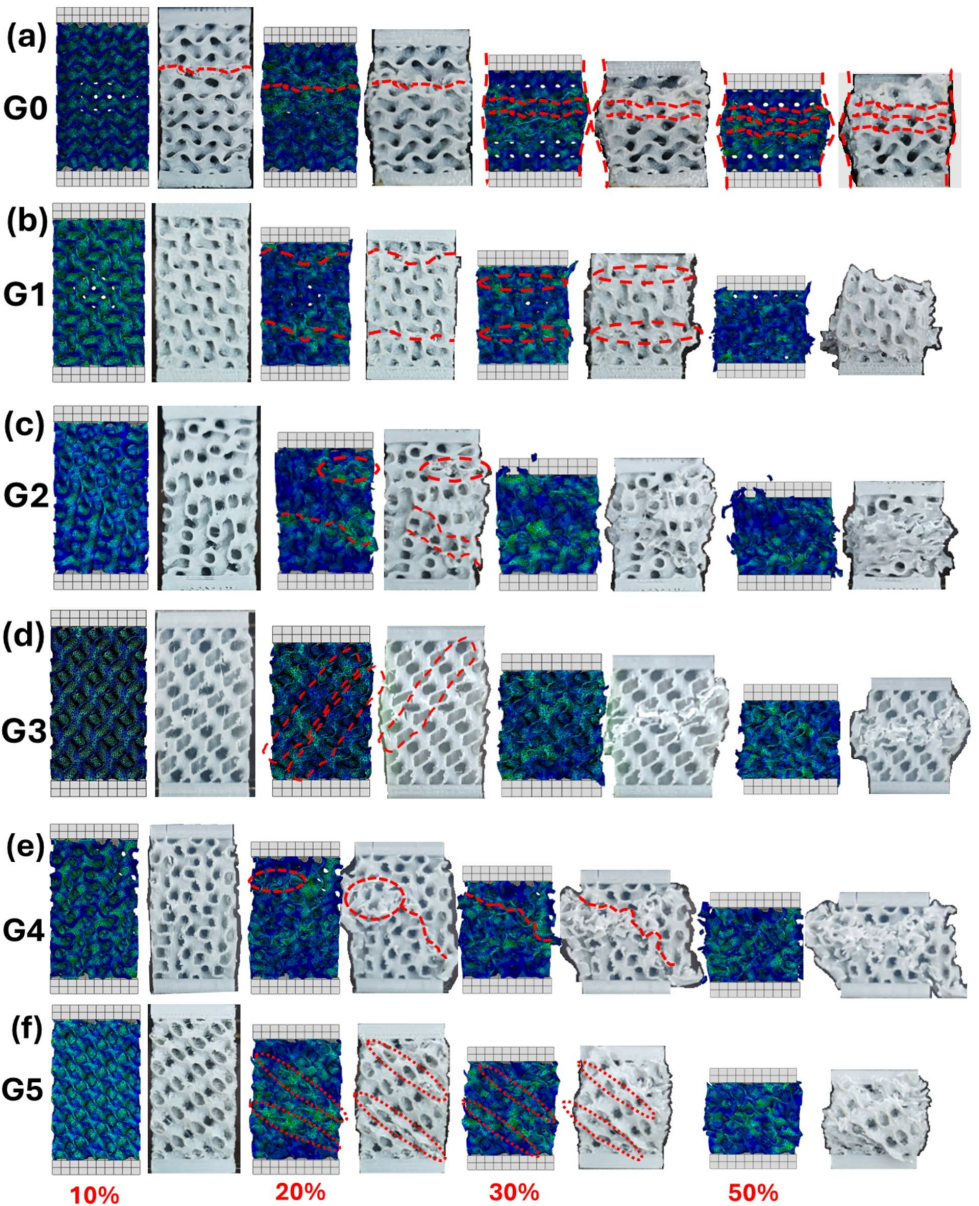
Progressive Compressive deformation and collapse behavior of TPMS Gyroid structures at nominal engineering strains of 10%, 20%, 30%, and 50%: (**a**) G0, (**b**) G1, (**c**) G2, (**d**) G3, (**e**) G4, and (**f**) G5.

The experimental and simulation compressive deformation and collapse behavior at progressive compressive strains (10%, 20%, 30%, and 50%) of different gyroid structures are shown in Fig. 14. Obviously, the collapse behavior aligns with the previously observed mechanical performance and supports the classification of deformation regimes depending on structural topology and strut orientation. In the G0 structure (Fig. 14a), deformation initiates with localized buckling at the mid-height region as early as 10–20% strain, with further loading resulting in the formation of a well-defined collapse band centered along the specimen axis. This is indicative of a bending-dominated mode, where structural instabilities emerge due to insufficient nodal connectivity and the lack of continuous load-bearing paths. The red dashed lines outlining the collapse region confirm the symmetry of failure and the evolution of plastic hinges, which is typical in open lattice designs. This behavior is consistent with G0’s low stiffness, yield strength, and peak stress reported earlier, confirming that the deformation energy is dissipated primarily through local flexural modes. Conversely, G1 exhibits a distinctly different behavior (Fig. 14b), with deformation distributed over multiple horizontal layers and progressing gradually through the height of the structure. At early strain levels (10–20%), axial deformation is accommodated uniformly, while at 30% and beyond, localized buckling appears in discrete horizontal bands. This suggests a progressive collapse mechanism governed by stretch-dominated response, where axial struts resist deformation until geometric instability is triggered. It is worth mentioning that the relatively high yield stress and plateau stress of G1, particularly at ~ 60% porosity (0.8 mm wall thickness), corroborate this interpretation. The dense and aligned gyroid structure in G1 provides effective load paths and delays catastrophic collapse, supporting its superior performance in both strength and energy absorption, as demonstrated in Fig. 10a-e.

In the G2 model (Fig. 14c), at 10% strain, minor structural distortion occurs, indicating early-stage elastic bending. By 20% strain, clear evidence of localized buckling and asymmetric deformation emerges-highlighted by the red dashed regions-where deformation initiates in weak zones lacking sufficient nodal reinforcement. This uneven strain localization results in partial layerwise collapse, suggesting a mixed-mode deformation dominated by bending with contributions from axial stretching. As shown in stress-strain results, G2 exhibits moderate E and σ_y_ values relative to G1 and G3 but higher than G0, reflecting its intermediary performance. As the strain progresses to 30% and 50%, failure becomes more disordered, with several strut breakages and collapse bands evident across the central height. The structural integrity deteriorates due to unstable load redistribution, confirming that G2 lacks the structural redundancy required for progressive densification under high strain, limiting its energy absorption capacity. In contrast, G3 (Fig. 14d) reveals a much more uniform and stable deformation profile. Across all strain levels (10% to 50%), the lattice exhibits layerwise compaction with no obvious signs of shear banding or asymmetric failure, suggesting a highly regular and isotropic load-bearing behavior. The FE results display consistent strain distribution with minimal concentration zones, implying that G3 maintains high structural integrity even under elevated strain. This performance directly correlates with highest mechanical performance across all porosity levels, particularly its high σ_y_ and σ_pl_ stresses, as previously observed in Fig. 11b, d. G3’s architectural uniformity promotes quasi-homogeneous axial deformation, characteristic of a well-balanced stretch-bending hybrid regime, enabling it to perform effectively under cyclic or dynamic loading conditions. The lack of catastrophic collapse or brittle fracture up to 50% strain further positions G3 as a structurally robust and energy-efficient design.

The G4 model (Fig. 14e) exhibits an intermediate deformation pattern. At 20% strain, strain localization emerges at random zones across the cross-section, indicating structural inhomogeneity in response to compressive loading. The red-marked failure zones suggest that crack initiation and propagation occur along paths of least resistance, possibly due to geometrical irregularities or weak nodal connections. At 30–50% strain, large portions of the structure collapse asymmetrically, forming a non-uniform shear failure pattern. These observations are in agreement with the moderate mechanical performance of G4 seen earlier, as its plateau stress and absorbed energy values are slightly lower than G1 and G5 despite comparable porosity levels, highlighting the sensitivity of gyroid performance to topology arrangement.

Particularly, G5 (Fig. 14f) demonstrates a pronounced diagonal shear banding failure mode, emerging at 20% strain and intensifying with further loading. The red dashed lines clearly depict a collapse mechanism dominated by shear-induced instability, which aligns with the alternating gyroid layer orientation. The oblique failure planes suggest a mode similar to shear band formation in metallic foams, where load is redirected along inclined paths due to structural anisotropy. The deformation is primarily concentrated in a single diagonl layer, and only when the corresponding layer was fully compressed another layer started to collapse. The full densification of these progressive layers reflected by a prominent peak in the stress-strain curves. Despite the brittle-looking final fracture appearance, G5 maintains relatively high energy absorption throughout the deformation. The diagonal shear pattern contributes to the load redistribution, sustaining mechanical integrity under large strains before complete collapse.

Hence, these deformation images reinforce the structure-property-processing relationship in architected models. G0’s bending-dominated collapse leads to early failure with minimal energy dissipation, while G1 and G5 exhibit stretch- and shear-dominated collapse, which supports high stress retention and progressive densification. G4’s performance sits in between, with behavior strongly influenced by internal irregularities and localized instabilities. The numerical model correlation with experimental validation enables a robust understanding of gyroid-based mechanical trends and confirms that geometrical design, governs deformation pathways and energy dissipation capacity.

### Mechanical properties-relative density relation analysis

To complement the qualitative analysis of the compression stress-strain behavior, the mechanical performance of the gyroid lattice structures (G0 to G5) was quantitatively evaluated using the Gibson-Ashby model, which captures the established dependency of mechanical properties on the relative density, ρ^−^ (ρ/ρs) of lattice structures through positive power-law relationships^49,64^. Normalized Young’s modulus E, yield stress σ_y,_ and absorbed energy W are utilized to compare and analyze the compressive properties of the TPMS structures, which are expressed as:

5 $$\:\frac{E}{{E}_{s}}={C}_{1}{\left(\frac{\rho\:}{{\rho\:}_{s}}\right)}^{{n}_{1}},$$

6 $$\:\frac{{\sigma\:}_{y}}{{\sigma\:}_{ys}}={C}_{2}{\left(\frac{\rho\:}{{\rho\:}_{s}}\right)}^{{n}_{2}},$$

7 $$\:\frac{W}{{\sigma\:}_{ys}}={C}_{3}{\left(\frac{\rho\:}{{\rho\:}_{s}}\right)}^{{n}_{3}}$$

where E_S_ and σ_y_ are Young’s modulus and yield strength of the bulk PLA. The coefficients C_1_; C_2_; C_3_ and exponents n_1_; n_2_; n_3_ differ for different TPMS geometries. These principles are well-established in literature and have been successfully applied to TPMS structures in prior studies^16,65^. The coefficients, exponents, and coefficients of determination (R²) obtained from power-law fitting of the mechanical properties for the various TPMS-based lattice structures are summarized in Table 4. The fitting results demonstrated strong agreement with the Gibson-Ashby scaling model, with R² values exceeding 0.95 for both the elastic modulus and the yield strength, and 0.97 for absorption energy, confirming the robustness of the model in capturing the mechanical behavior of the structures.

**Table 4:** Coefficients of power fitting analysis for mechanical properties of different G-models.

Model	E/Es			σ_y_/σ_ys_			W/σ_ys_		
	C_1_	*n*_1_	*R* ^2^	C_2_	*n*_2_	*R* ^2^	C_3_	*n*_3_	*R* ^2^
G0	0.15	0.61	0.98	0.13	0.45	0.95	0.06	0.50	0.96
G1	0.40	0.77	0.99	0.36	0.81	0.98	0.15	1.09	0.99
G2	0.68	1.39	0.99	0.45	1.04	0.98	0.16	1.15	0.99
G3	0.49	1.05	0.97	0.41	0.86	0.97	0.10	0.70	0.96
G4	0.38	0.78	0.95	0.36	0.82	0.95	0.11	0.72	0.97
G5	0.53	1.21	0.96	0.54	1.20	0.99	0.19	1.26	0.99

According to the Gibson-Ashby, the mechanical properties of lattice structures are closely influenced by their dominant deformation mode bending or stretch-dominated. For bending-dominated lattices, the relative elastic modulus scales with the square of the relative density (n_1_ = 2), and the relative yield strength scales with the 1.5 power of the relative density (n_2_ = 1.5), both characterized by a proportionality constant *C₁* ^49^. In contrast, for stretch-dominated structures, both the relative modulus and strength exhibit a linear relationship with relative density (n_1_ = n_2_ = 1), governed by a different constant *C₂*^64^. Moreover, a more recent study identified bending-dominated lattice structures and proposed a narrower and more precise range for the proportionality constants compared to those originally suggested by the Gibson-Ashby model. In that study, C₁ for metallic open-celled bending-dominated lattices was reported to lie within the range 0.1–0.6, while C₂ remained consistent with the values predicted by the original model.

**Fig. 15:**
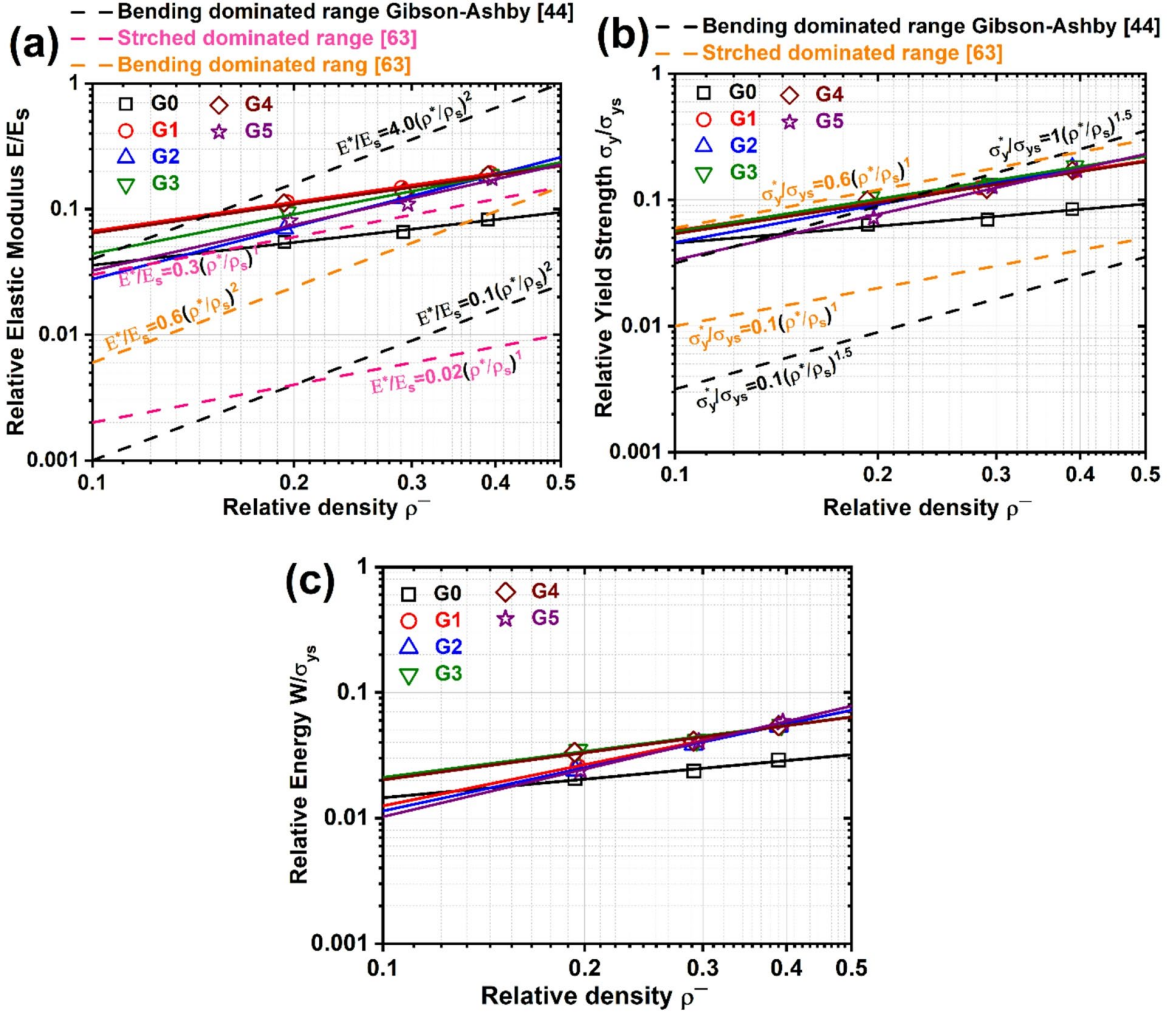
Log–log plots of power laws relating the: (**a**) relative elastic modulus and (**b**) relative yield strength, (**c**) relative energy of the Gyroid lattice structures to the relative density, and the range for bending-dominated and stretch-dominated structures stated by ^51,70^.

For relative modulus (Fig. 15a), the fitted exponents suggest a mixed-mode behavior, with deformation mechanisms involving both bending and stretching. G1-G5 follow closely aligned power-law trends, positioned between the classic stretch- and bending-dominated regimes, except G1 and G4, implying a semi-stiff response that balances energy absorption and stiffness. The fitted curves fall largely within or just above the bending-dominated range identified by Almor et al. ^66^, rather than matching the original Gibson-Ashby upper limit.

The relatively higher slopes confirm that elastic deformation in these structures is primarily governed by bending, particularly at lower relative densities. However, as density increases, certain topologies, most notably G5, begin to approach the upper bound, indicating a transition toward stretch-dominated behavior. This is likely due to increased load path connectivity and geometric constraints. For instance, based on the fitted equations, the compressive modulus of G5 is approximately 11% lower than that of G0 at a ρ^−^ of 0.1. In contrast, at a ρ^−^ of 0.4, G5 demonstrates a 99% increase in modulus over G0, indicating a significant stiffness enhancement at higher densities. Similarly, G1 and G4 exhibit modulus improvements of approximately 70% and 24%, respectively, compared to G0 at ρ/ρs = 0.1 and 0.4. These results emphasize that the designed topology enables a tunable mechanical response, offering a wide range of stiffness and deformation characteristics depending on the intended application.

The relative yield strength of the lattice structures is accurately predicted within the predefined bounds for stretch-dominated behavior (Fig. 15b). All models lie within the range specified by Alomar et al. for stretch‐dominated structures, while remaining well above the Gibson-Ashby bending‐dominated limits. Practically, this indicates that even very lightweight gyroids derive most of their initial stiffness and strength from axial strut action rather than bending. One contributing factor is that Gibson-Ashby proportionality constants were derived from experimental data, whereas this study uses ideal CAD models; in reality, additively manufactured lattices exhibit reduced elastic moduli due to defects such as partially melted powder particles and residual thermal stresses. Particularly, at the lowest relative density, G5 exhibits the smallest yield strength, but at the highest density it reaches a peak that is 146% higher than G0. At intermediate densities, G3 achieves the highest yield strength, with increases of 63%, 92%, and 116% over G0 at relative densities of 0.20, 0.30, and 0.40, respectively. Oriented gyroid variants (G1-G5) achieve higher yield strength than the base model G0 because rotating the minimal‐surface network aligns more ligaments with the loading axis, increasing their axial load‐bearing fraction and reducing bending moments. This alignment shortens unsupported strut lengths, raising the critical buckling stress, and creates more direct vertical load paths, thereby delaying local yielding.

Across the tested density range, all gyroid variants show a clear upward trend in normalized energy absorption, specifically at greater relative density (ρ^−^ > 0.2), as shown in Fig. 15c. This behavior mirrors previously reported gyroid studies, which found that aligning ligaments closer to the loading axis not only boosts yield strength but also enhances progressive collapse and energy dissipation. Orienting the TPMS increases the number of struts that deform axially rather than by bending during compaction, thereby extending the plateau region of quasi-plastic collapse and raising the total energy absorbed before densification.

**Fig. 16:**
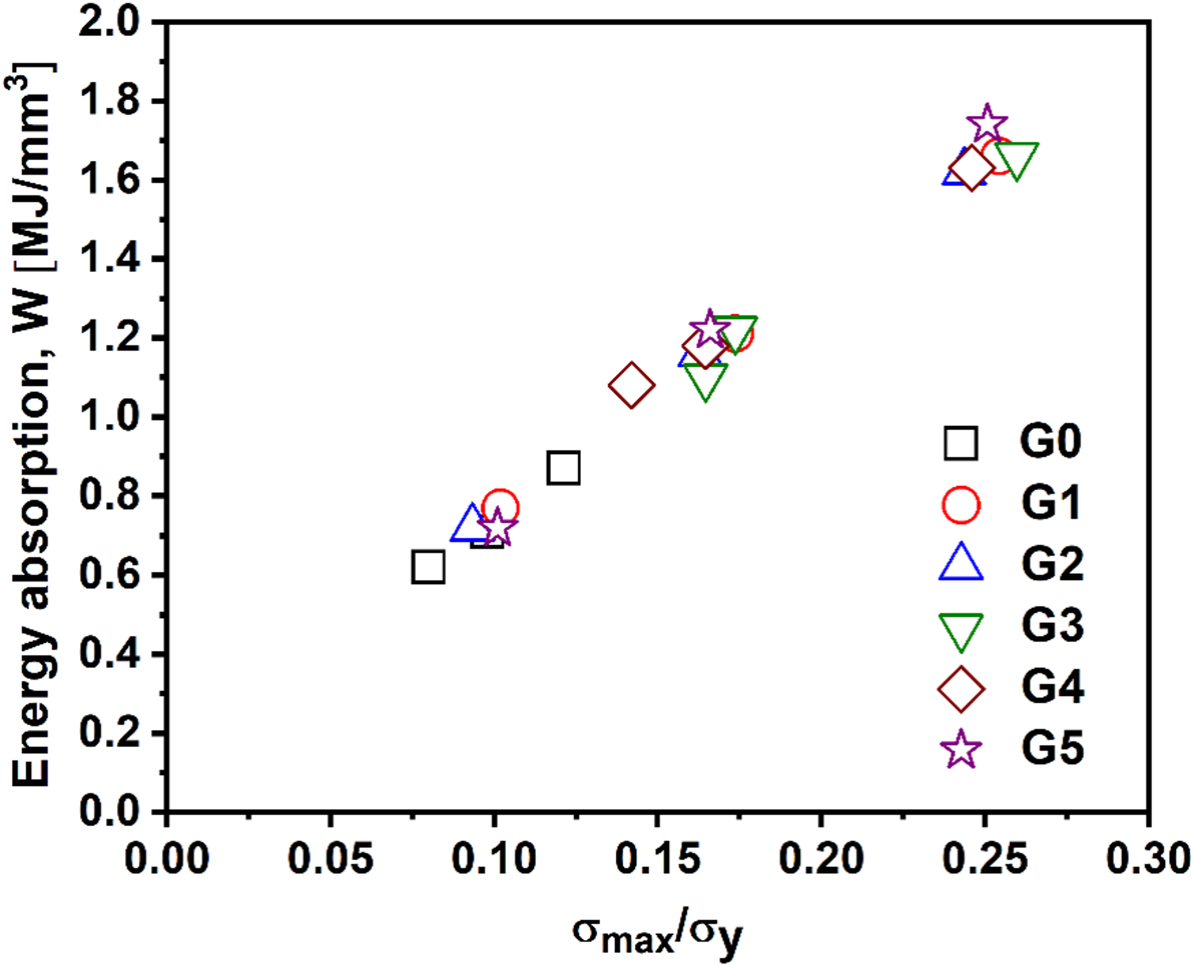
Correlation between absorption energy (W) and normalized maximum stress (σ_max_/σ_y_) for TPMS lattice structures (G0–G5).

Figure 16 illustrates the relationship between energy absorption and the normalized maximum stress (σ_max_/σ_y_) for the gyroid lattice structures G0 to G5. As earlier show, absorption energy, which increases with relative density, is best observed as long as the maximum conducted stress, is not just the initial maximum stress, but the true maximum during the portion of the stress-strain response utilized in the energy calculation^67^. Following this approach, absorption energy vs. the normalized maximum stress (divided by PLA yield strength) is shown in Fig. 16. G0 lies in the lower-left corner, with the lowest stress ratio and energy absorption, indicating limited load-bearing and poor energy dissipation. This is consistent with its minimal material distribution and bending-dominated deformation under compression. G5 and G3 occupy the upper-right region, signifying the highest energy absorption and largest σ_max_/σ_y_ ratios, suggesting that these structures exhibit stable, progressive deformation and delayed yielding, key for energy-absorbing applications. G5 stands out with the highest values, aligning with earlier findings that its performance improves significantly at higher relative densities due to its uniform material distribution and enhanced load-path connectivity. Hence, such behavior represents an ideal candidate for impact mitigation, protective structures, or bio-inspired load-damping systems, where controlled deformation and effective energy dissipation are critical.

**Fig. 17:**
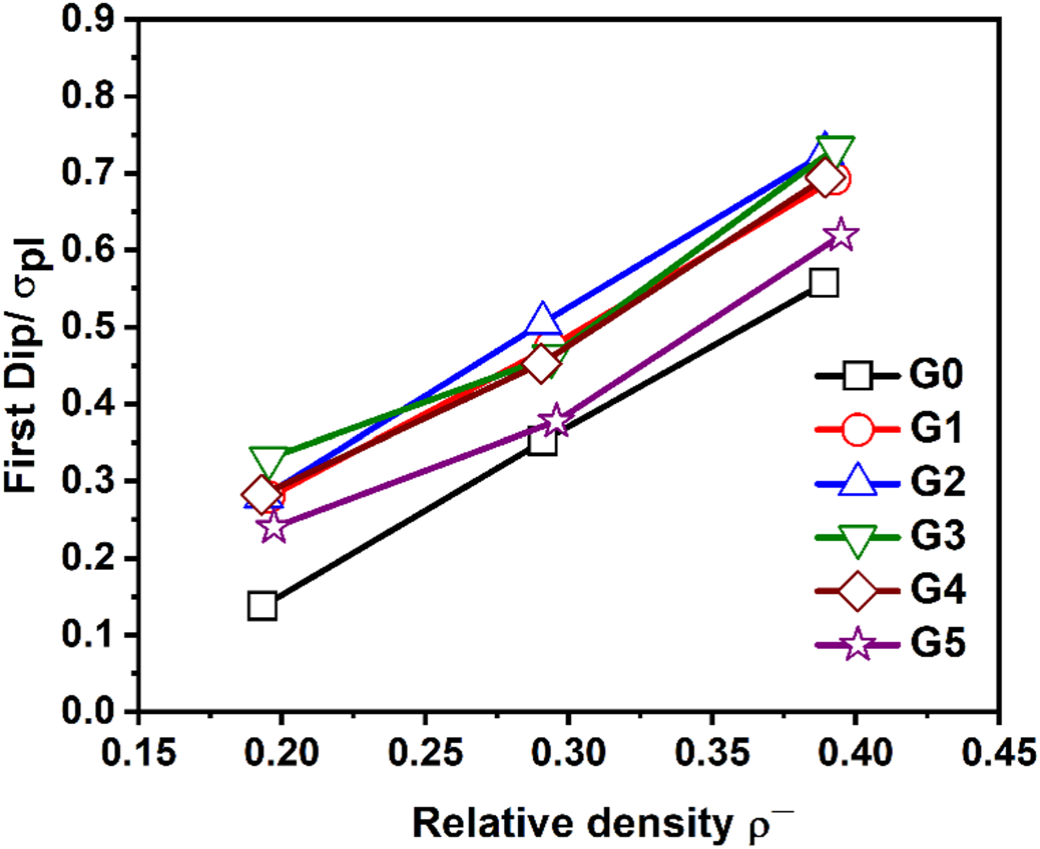
Variation of the normalized first stress dip (First Dip/σ_pl_) as a function of relative density (ρ/ρ_s_) for gyroid lattice structures G0-G5.

Undulations in the stress plateau have the undesirable effect of limiting absorption energy. In this manner, Fig. 17 illustrates the variation of the normalized first stress dip (First Dip/σₚₗ) as a function of relative density for the six TPMS lattice structures. The “first dip” refers to the initial post-yield stress drop that occurs after the elastic peak and before the onset of the plateau region in the stress-strain curve. It reflects early structural instability or collapses behavior under compressive loading. At lower densities (~ 0.2–0.3), G5 consistently exhibits the lowest normalized first dip, suggesting greater early instability, likely due to its more uniform cross-sectional area, which may reduce initial load path redundancy. In contrast, model G2, G3 and G4 show higher First Dip/σ_pl_ values across all densities, indicating more stable collapse behavior and earlier engagement of the plateau regime, which is favorable for energy-absorbing applications. Hence, topologies like G3 and G4 offer a favorable balance between structural integrity and energy dissipation, while G5, despite its high overall performance, may be prone to early local collapse at lower densities.

## Conclusion

This study comprehensively examined the mechanical performance and energy absorption behaviour of gyroid-based Triply Periodic Minimal Surface (TPMS) lattice structures, focusing on the effects of build orientation and wall thickness through both experimental testing and finite element modelling. The key findings are summarised below:

- Finite Element Analysis (FEA) showed strong agreement with experimental data, validating the numerical approach for accurately predicting deformation behavior and supporting the optimization of TPMS-based designs.
- Gyroid structures with axially aligned struts at 30°, 90°, and 180° (G1, G3, G5, respectively) exhibited significantly enhanced stiffness, yield strength, and energy absorption, highlighting the critical role of orientation in optimizing load paths.
- A transition from bending- to stretch-dominated deformation was observed with increasing relative density, especially in the 90° (G3) and 180° (G5) oriented models, leading to improved load-bearing capacity and energy dissipation.
- The Gibson-Ashby power-law model accurately captured the mechanical property-density relationship, with high correlation (R² > 0.95), reinforcing its applicability to orientation-optimized TPMS lattices.
- Gyroid models oriented at 90° and 180° (G3 and G5) demonstrated exceptional energy absorption, indicating their suitability for impact-sensitive applications in automotive safety, aerospace structures, and biomedical implants.
- Despite strong overall performance, highly uniform topologies such as G5 at 180° showed early localized instabilities at lower densities, emphasizing the need to balance energy absorption with structural stability in lightweight design.

Overall, this work emphasises the significance of geometric orientation and relative density in tailoring the mechanical behaviour of TPMS lattices, providing a solid foundation for developing next-generation architected materials across diverse engineering applications.

## Data Availability

All the raw data supporting the conclusion of this paper was provided by the authors on request.
